# Potential‐Modulated Ion Distributions in the Back‐to‐Back Electrical Double Layers at a Polarised Liquid|Liquid Interface Regulate the Kinetics of Interfacial Electron Transfer

**DOI:** 10.1002/celc.202201042

**Published:** 2022-12-27

**Authors:** Alonso Gamero‐Quijano, José A. Manzanares, Seyed M. B. H. Ghazvini, Paul J. Low, Micheál D. Scanlon

**Affiliations:** ^1^ Department of Physical Chemistry University of Alicante (UA) E-03080 Alicante Spain; ^2^ The Bernal Institute and Department of Chemical Sciences School of Natural Sciences University of Limerick (UL) Limerick V94 T9PX Ireland; ^3^ Department of Thermodynamics Faculty of Physics University of Valencia c/Dr. Moliner, 50 Burjasot E-46100 Valencia Spain; ^4^ School of Molecular Sciences University of Western Australia (UWA) 35 Stirling Highway Crawley Western Australia 6009 Australia

**Keywords:** interface between two immiscible electrolyte solutions (ITIES), interfacial electron transfer, oxygen reduction reaction, polarised liquid|liquid interface, potential of zero charge (PZC)

## Abstract

Biphasic interfacial electron transfer (IET) reactions at polarisable liquid|liquid (L|L) interfaces underpin new approaches to electrosynthesis, redox electrocatalysis, bioelectrochemistry and artificial photosynthesis. Herein, using cyclic and alternating current voltammetry, we demonstrate that under certain experimental conditions, the biphasic 2‐electron O_2_ reduction reaction can proceed by single‐step IET between a reductant in the organic phase, decamethylferrocene, and interfacial protons in the presence of O_2_. Using this biphasic system, we demonstrate that the applied interfacial Galvani potential difference $\Delta_{o}^{w}\varphi$ provides no direct driving force to realise a thermodynamically uphill biphasic IET reaction in the mixed solvent region. We show that the onset potential for a biphasic single‐step IET reaction does not correlate with the thermodynamically predicted standard Galvani IET potential and is instead closely correlated with the potential of zero charge at a polarised L|L interface. We outline that the applied $\Delta_{o}^{w}\varphi$ required to modulate the interfacial ion distributions, and thus kinetics of IET, must be optimised to ensure that the aqueous and organic redox species are present in substantial concentrations at the L|L interface simultaneously in order to react.

## Graphical abstract

**Biphasic interfacial electron transfer**: Using the biphasic 2‐electron O_2_ reduction reaction at a polarised liquid|liquid interface, the applied interfacial Galvani potential difference is shown to provide no direct driving force to realise a thermodynamically uphill interfacial electron transfer (IET) reaction. Thermodynamically spontaneous IET is kinetically inhibited until is tuned to modulate the ion distributions such that all species involved in the biphasic reaction are present at the interface in significant concentrations simultaneously.

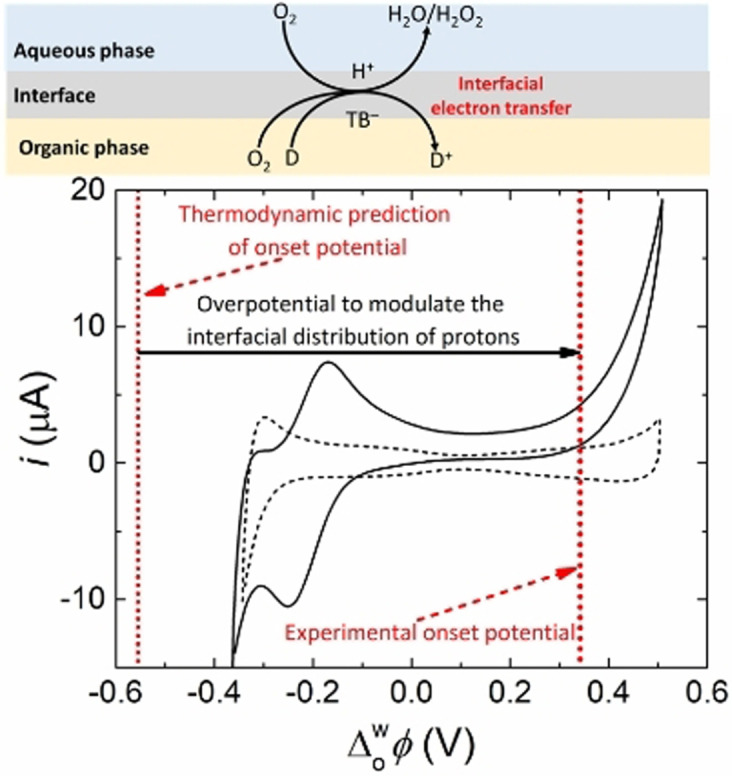

## Introduction

A comprehensive understanding of biphasic interfacial electron transfer (IET) reactions between aqueous and organic soluble redox couples at the interface between two immiscible electrolyte solutions (ITIES) provides the fundamental foundation on which an ever‐increasing range of applications are based, such as: (i) interfacial electrosynthesis of thin films of advanced functional materials, e. g., conducting polymers;^[1]^ (ii) interfacial redox electrocatalysis of energy conversion and storage (ECS) reactions, e. g., the biphasic H_2_ evolution reaction (HER), O_2_ reduction reaction (ORR) and O_2_ evolution reaction (OER);[^2^, ^3^, ^4^] (iii) interfacial bioelectrochemistry of proteins, e. g., cytochrome *c*, to replicate the molecular machinery of biomembranes;[^5^, ^6^] and (iv) interfacial photoconversion reactions involving porphyrins towards artificial photosynthesis.[^7^, ^8^, ^9^, ^10^]

The modified Verwey‐Niessen (MVN) model describes the nature of the electric double layer (EDL) at the ITIES.[^11^, ^12^] The latter consists of a mixed solvent region, which can be penetrated partially by ions from both phases, separating two back‐to‐back diffuse EDLs. The mixed solvent region is approximately 1 nm thick for commonly studied polarisable liquid|liquid (L|L) interfaces, such as those formed between aqueous electrolyte solutions and organic electrolyte solutions prepared with the solvents 1,2‐dichloroethane (DCE) or α,α,α‐trifluorotoluene (TFT).^[13]^ The interfacial Galvani potential difference $\Delta_{o}^{w}\varphi$ applied at a polarisable L|L interface can be: (i) varied dynamically by employing a 4‐electrode electrochemical cell and implementing standard electrochemical techniques, such as cyclic voltammetry (CV) or alternating current voltammetry, using a potentiostat;^[14]^ or (ii) set to a single value by distribution of a common ion or salt between the phases.^[15]^ The applied $\Delta_{o}^{w}\varphi$ drops across the two back‐to‐back EDLs, with the majority dropping on the organic side of the EDL,^[16]^ as explained in detail *vide infra*. For dilute electrolyte concentrations, the Debye lengths associated with the back‐to‐back EDLs substantially exceed the 1 nm width of the mixed solvent region.^[17]^ Thus, the magnitude of the potential drop within the mixed solvent region is relatively minor compared to the applied $\Delta_{o}^{w}\varphi$ and is negligible at the potential of zero charge (PZC).^[18]^

As initially described by Girault and Schiffrin,^[19]^ for a biphasic single‐step IET reaction to proceed, an interfacial precursor must form prior to the charge transfer step within the mixed solvent region. In this model, variation of the applied $\Delta_{o}^{w}\varphi$ can affect the kinetics of IET by: (i) changing the Gibbs free energy between the different redox species participating in the interfacial precursor; and (ii) providing a driving force to bring redox reactants from the bulk through the diffuse EDLs to the mixed solvent region to form the precursor. However, as a biphasic single‐step IET reaction proceeds exclusively in the mixed solvent region where the potential drop experienced is minor, the Gibbs free energy between the two redox species in the precursor is unaffected by the applied $\Delta_{o}^{w}\varphi$ . Consequently, analysis of biphasic single‐step IET reactions using classical theory developed to explain electron transfer reactions at solid electrode|electrolyte interfaces, such as Butler‐Volmer kinetics, is invalid.[^17^, ^20^, ^21^] Instead, the kinetics of a biphasic single‐step IET reaction is primarily influenced by the changes in concentration of the redox species on either side of the L|L interface as a function of the applied $\Delta_{o}^{w}\varphi$ .

In this article, using the biphasic 2e^−^ ORR with a series of ferrocene derivatives as the reductants, we demonstrate that only biphasic single‐step IET reactions that are mechanistically feasible and thermodynamically spontaneous in the mixed solvent region may lead to an observable IET signal within the polarisable potential window (PPW) at a polarised L|L interface. We find that the onset potential for a biphasic single‐step IET reaction ($\Delta_{o}^{w}\varphi_{IET}^{onset}$ ) does not correlate with the thermodynamically predicted standard Galvani IET potential $\left(\Delta_{o}^{w}\varphi_{IET}^{0}\;\right)$ , and is instead closely correlated with the PZC at a polarised L|L interface. In this regard, using a Verwey‐Niessen model of the polarised L|L interface, we calculate the interfacial concentrations of ions that accumulate in the back‐to‐back EDLs upon polarisation of the L|L interface as a function of the applied $\Delta_{o}^{w}\varphi$ . A general discussion is provided to explore the influence of the nine possible ionic distributions of the redox reactants (which can be either cationic, anionic or neutral) on either side of the L|L interface as a function of the applied $\Delta_{o}^{w}\varphi$ prior to the biphasic single‐step IET reaction. Finally, we discuss that under certain circumstances, the applied $\Delta_{o}^{w}\varphi$ can *indirectly* drive a biphasic single‐step IET reaction predicted to be thermodynamically uphill based on the standard redox potentials $E^{0}$ of the aqueous and organic redox species in their respective bulk phase.

## Results and Discussion

### Methodologies employed to realise the biphasic ORR at a polarised L|L interface

**Scheme 1:**
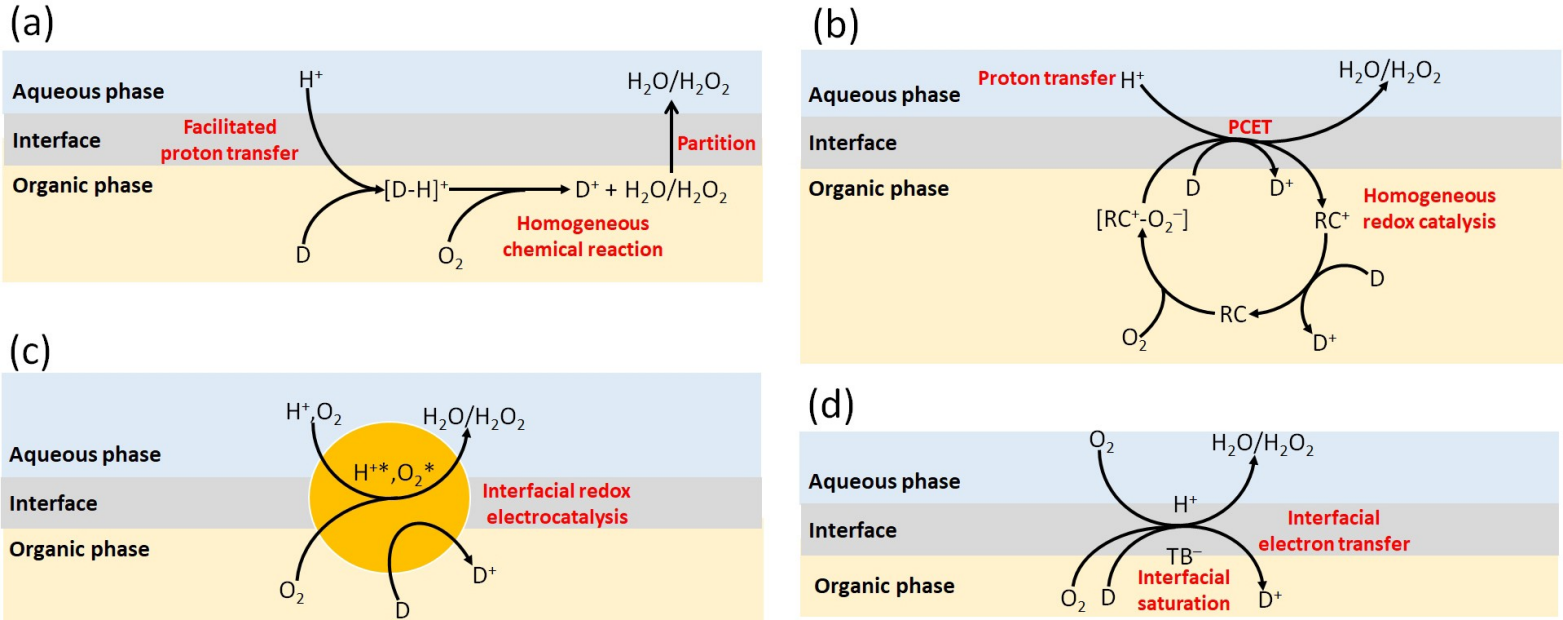
Schematic of the different pathways to achieve the biphasic ORR at a polarised L|L interface. (a) Facilitated proton transfer coupled with a homogeneous chemical reaction. D is an electron donor, typically a metallocene such as decamethylferrocene and its derivatives or tetrathiafulvalene. [D−H]^+^ is an intermediate hydride species formed between the proton and electron donor. (b) Interfacial molecular electrocatalysis involving facilitated proton transfer coupled to homogeneous redox catalysis *via* a proton‐coupled electron transfer (PCET) step. RC is a redox catalyst present in the organic phase that binds O_2_, typically a molecular species such as a free‐base or metallo porphyrin/porphine/phthalocyanine. (c) Interfacial redox electrocatalysis whereby an interfacially adsorbed conductive solid (nano)material, such as metallic nanoparticles and carbon nanomaterials, facilitates the flow of electrons and Fermi level equilibration between the aqueous proton and organic electron donor redox couples. H^+^* and O_2_* are protons and O_2_ adsorbed on the redox electrocatalysts surface. (d) Single‐step interfacial electron transfer involving interfacial saturation of the organic electrolyte anion, tetrakis(pentafluorophenylborate) (TB^−^), in the electric double layer (EDL) on the organic side of the L|L interface. This build‐up of negative charge on the organic side of the L|L interface is compensated by an accumulation of aqueous cations (e. g., protons) in, and repulsion of aqueous anions (e. g., sulfate anions) from, the EDL on the aqueous side of the L|L interface such that the charge densities in the back‐to‐back EDLs of opposite sign compensate each other ($Q^{w}=-Q^{o}$ ). For all pathways described, the biphasic ORR requires the application of $\Delta_{o}^{w}\varphi$ positive of the potential of zero charge.

As reviewed in detail recently by Opallo et al.,^[3]^ three distinct methodologies have been employed to date to realise the biphasic ORR using lipophilic ferrocene derivatives as electron donors at a polarised L|L interface formed between immiscible aqueous and organic electrolyte solutions. The first methodology involves scanning the applied $\Delta_{o}^{w}\varphi$ to the positive edge of the PPW to initiate the transfer of aqueous protons (H_3_O^+^) facilitated by a ferrocene derivative, such as decamethylferrocene (DcMFc)^[22]^ or 1,2‐diferrocenylethane,^[23]^ from an acidic aqueous phase to the organic phase (Scheme 1a). This initial electrochemical facilitated ion transfer step is followed by a homogeneous chemical reaction in the organic phase where a proton binds to the metal centre of the ferrocene derivative forming a hydride species. The hydride subsequently reacts with dissolved O_2_, yielding the oxidised ferrocene derivative and H_2_O_2_ *via* a hydrogen peroxyl radical intermediate. A similar mechanism can operate under non‐acidic conditions, whereby a metal cation (Li^+^, Na^+^, K^+^) undergoes ion transfer to the organic phase with its hydration shell at least partially intact at an applied $\Delta_{o}^{w}\varphi$ at the positive edge of the PPW.[^24^, ^25^] Subsequently, in the organic phase, the metal cation behaves as a Lewis acid, coordinating to the oxygen of the water molecules surrounding it in the hydration shell, weakening the O−H bonds and, thus, activating these water molecules as the proton source to form a hydride species with the ferrocene derivative.

The second methodology, interfacial molecular electrocatalysis, has been achieved by introducing a homogeneous redox catalyst, such as a metallo‐ (Co^(II)^ or Fe^(II)^) or free‐base porphyrin,[^26^, ^27^, ^28^, ^29^, ^30^] porphine^[31]^ or phthalocyanine,[^32^, ^33^, ^34^] to the organic phase (Scheme 1b). The use of a metallo‐redox catalyst activates O_2_ towards reduction by relatively weak reductants, e. g., dimethylferrocene (DiMFc) and ferrocene (Fc), *via* coordination with the metal centre. The O_2_‐redox catalyst complex facilitates the simultaneous ion transfer of H_3_O^+^ from the aqueous phase and electron transfer from the ferrocene derivative in a proton‐coupled electron transfer (PCET) step. Furthermore, using cofacial “Pacman” Co^(II)^ porphyrins allows the selectivity of the biphasic ORR to shift from a 2e^−^ mechanism that yields H_2_O_2_ as the primary product, as is the case in the absence of a redox catalyst, to a 4e^−^ mechanism yielding water.^[27]^

The third methodology, interfacial redox electrocatalysis, involves the catalysis of biphasic IET between two redox couples using a floating conductive catalyst at a polarised L|L interface.[^4^, ^35^] Using this methodology (Scheme 1c), the biphasic ORR has been catalysed by gold (Au),[^36^, ^37^] platinum (Pt),[^38^, ^39^] and gold‐palladium (Pd@Au) core‐shell nanoparticles,^[36]^ as well as carbon‐based nanomaterials (reduced graphene oxide,^[40]^ few‐layer graphene^[41]^ and lithium‐ion battery waste^[42]^). The mechanism involves the floating conductive catalyst providing a catalytic surface for the reactants to adsorb onto (enhancing the kinetics of one or both half‐reactions) and acting as a bipolar electrode to facilitate catalysis through Fermi level equilibration by direct IET between the redox couples (providing an additional thermodynamic driving force). In other words, the ferrocene derivative can “charge” the floating conductive catalyst on the organic side of the L|L interface, with the “discharge” reaction being the ORR on the aqueous side. The position of the Fermi level in the floating conductive catalyst is dictated by the relative kinetics of the charge and discharge processes and the applied $\Delta_{o}^{w}\varphi$ , and the extra thermodynamic driving force provided enables the biphasic ORR to proceed using the relatively weak reductants DiMFc^[36]^ and Fc.[^38^, ^40^, ^43^] Furthermore, owing to the Fermi level equilibration process, the O_2_, protons and ferrocene derivative do not need to meet simultaneously in the mixed solvent region to react.

Each of the three methodologies to achieve the biphasic ORR described above are multi‐step, involving facilitated ion transfer, O_2_ activation by a metallo‐redox catalyst or Fermi‐level equilibration steps prior to IET. In this article, we introduce a fourth methodology, which is simply direct or single‐step IET between O_2_, protons and a suitable ferrocene derivative (identified as DcMFc, as discussed in detail *vide infra*) in the mixed solvent region at the polarised L|L interface (Scheme 1d). The key difference between this approach and the first methodology described is that the applied $\Delta_{o}^{w}\varphi$ is not scanned to positive values sufficient to initiate the transfer of protons facilitated by DcMFc or the transfer of hydrated metal cations (such as Li^+^) to the organic phase. Instead, the applied $\Delta_{o}^{w}\varphi$ is carefully reversed once sufficiently positive of the PZC (usually by ca. 300 mV) to “saturate” the mixed solvent region with protons. The interfacial concentration of protons is limited to the positive charge required to compensate the simultaneous accumulation of the organic electrolyte anion tetrakis(pentafluorophenylborate) (TB^−^) anions in the mixed solvent region during polarisation of the L|L interface.

### Thermodynamics of biphasic ORRs and HERs at a polarised L|L interface

In this section, the feasibility of electrochemically observing biphasic ORRs and HERs within the PPW at a polarised aqueous|TFT interface via a single‐step IET mechanism as described in Scheme 1d is explored from a purely thermodynamic viewpoint. The reactant O_2_ may be supplied for biphasic ORRs either from the aqueous or organic phase and in principle can undergo either a 2e^−^ or 4e^−^ ORR or form perhydroxyl radicals (HO_2_⋅) or superoxide radical anions (O_2_⋅^−^) (Table S1). Due to the absence of protons in the organic phase, these heterogeneous ORRs are assumed to only take place with aqueous protons (H_3_O^+^) within ∼10 nm of the L|L interface or in the mixed solvent region involving accumulated protons in the EDL on the aqueous side of the L|L interface that compensate the charge of the organic electrolyte TB^−^ anions in the EDL on the organic side of the L|L interface ([H^+^…TB^−^]).

**Figure 1:**
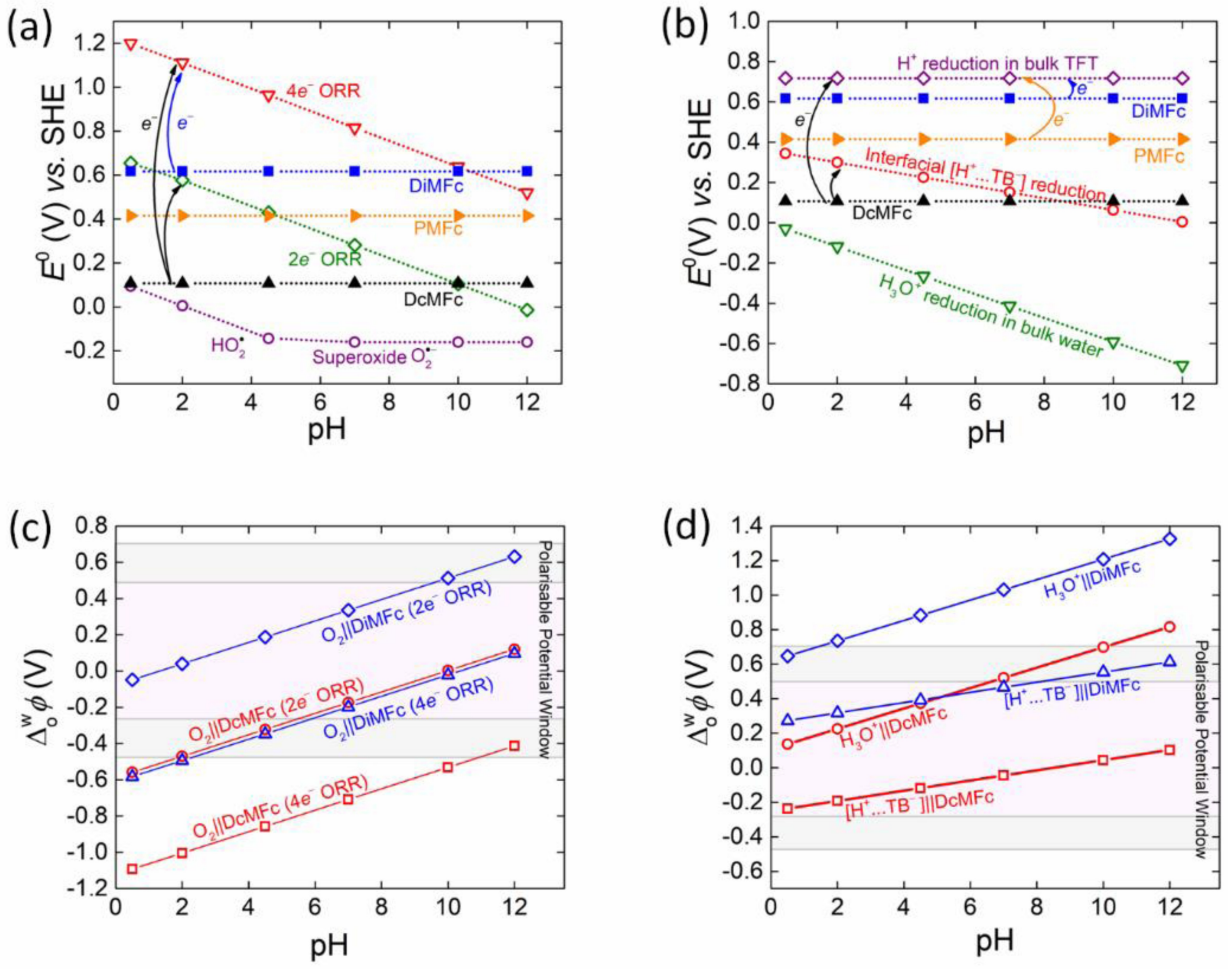
The thermodynamics of biphasic molecular oxygen (O_2_) reduction and biphasic proton reduction with organic solubilised electron donors at a polarised liquid|liquid (L|L) interface. (a) Plots of the standard redox potentials *E* ^0^ vs. the standard hydrogen electrode (SHE) as a function of pH for (i) O_2_ undergoing either a 2e^−^ or 4e^−^ O_2_ reduction reaction (ORR) or forming perhydroxyl radicals (HO_2_⋅) or superoxide radical anions (O_2_⋅^−^) in a bulk aqueous solution, and (ii) the hydrophobic electron donors decamethylferrocene (DcMFc), pentamethylferrocene (PMFc) and dimethylferrocene (DiMFc) in the organic solvent α,α,α‐trifluorotoluene (TFT). (b) Plots of *E* ^0^ vs. SHE as a function of pH for (i) the reduction of protons to molecular hydrogen (H_2_) for protons in an aqueous solution (H_3_O^+^), organic TFT solution (H^+^), and accumulated in the EDL on the aqueous side of the L|L interface to compensate the charge of the organic electrolyte TB^−^ anions ([H^+^…TB^−^]) in the mixed solvent region, and (ii) DcMFc, PMFc and DiMFc in TFT. (c) Plots of the standard Galvani interfacial electron transfer (IET) potential $\left(\Delta_{o}^{w}\varphi_{IET}^{0}\;\right)\;$ as a function of pH for the biphasic ORR (2e^−^ or 4e^−^ pathways) with DcMFc and DiMFc as the organic electron donors, respectively. For clarity the corresponding plot with PMFc is provided in Figure S2a. (d) Plots of $\Delta_{o}^{w}\varphi_{IET}^{0}$ as a function of pH for biphasic reduction of H_3_O^+^ and interfacial [H^+^…TB^−^] with DcMFc and DiMFc as the organic electron donors, respectively. For clarity the corresponding plot with PMFc is provided in Figure S2b. In (c) and (d), the grey shaded areas of the Galvani polarisable potential window (PPW) represent applied interfacial Galvani potential differences ($\Delta_{o}^{w}\varphi$ ) at the positive and negative extremes of the PPW that lead to significant changes in the polarity, viscosity, relative permittivity, and interfacial tension of the polarised L|L interface. The sources of electrochemical data and equations used to construct plots (a) and (b) are outlined in Table S1, plot (c) in Table S2 and plot (d) in Table S3.

The standard redox potentials *E* ^0^ vs. the standard hydrogen electrode (SHE) as a function of pH for each possible ORR and HER redox couple are compared with the *E* ^0^ values of a range of different hydrophobic organic electron donors decamethylferrocene (DcMFc), pentamethylferrocene (PMFc) and 1,1’‐dimethylferrocene (DiMFc) in α, α, α ‐trifluorotoluene TFT in Figures 1a and b, respectively. The *E* ^0^ values of PMFc and DiMFc in TFT *vs*. SHE were determined using a three‐electrode electrochemical cell with the DcMFc^+^/DcMFc redox couple in TFT (*E* ^0^=+0.107 V vs. SHE) as an internal redox standard (Figure S1). The plots in Figures 1a and b allow an initial identification of which biphasic ORRs and HERs may be thermodynamically spontaneous as a function of pH via single‐step IET with each ferrocene derivative. IET only proceeds spontaneously (represented by curved arrows in Figures 1a and b) when *E* ^0^ of the organic electron donor redox couple, e. g., ${\left[E_{{DcMFc}^{+}/DcMFc}^{0}\right]}_{SHE}^{TFT}$ , is less positive than *E* ^0^ of the aqueous acceptor redox couple, e. g., ${\left[E_{O_{2}/H_{2}O_{2}}^{0}\right]}_{SHE}^{aq}$ for the 2e^−^ ORR. The latter means that the standard Galvani IET potential $\Delta_{o}^{w}\varphi_{IET}^{0}\;$ is less than 0 V, Equation 1:

(1) $$\Delta_{o}^{w}\varphi_{IET}^{0}={\left[E_{{Ox}_{1}/{Red}_{1}}^{0}\right]}_{SHE}^{TFT}-{\left[E_{{Ox}_{2}/{Red}_{2}}^{0}\right]}_{SHE}^{aq}\;.$$

The value $\Delta_{o}^{w}\varphi_{IET}^{0}=0$ defines the equilibrium point of a reversible biphasic IET reaction. Negative values on the Galvani scale ($\Delta_{o}^{w}\varphi_{IET}^{0}<0\;\;V)$ accelerate electron transfer from an organic to aqueous redox couple (as is the case discussed in this article). Positive values on the Galvani scale ($\Delta_{o}^{w}\varphi_{IET}^{0}>0\;\;V)$ favour electron transfer in the opposite direction, from an aqueous to organic redox couple. The standard Gibbs energy of IET when the direction of electron flow is from an organic to an aqueous redox couple (${\Delta_{o}^{w}G}_{IET}^{0}$ ) or aqueous to organic redox couple (${\Delta_{w}^{o}G}_{IET}^{0}$ ) is defined by Equations (2) and (3), respectively:

(2) $${\Delta_{o}^{w}G}_{IET}^{0}=nF\Delta_{o}^{w}\varphi_{IET}^{0}$$

(3) $${\Delta_{w}^{o}G}_{IET}^{0}=-nF\Delta_{o}^{w}\varphi_{IET}^{0}$$

where *n* is the number of electrons transferred during the biphasic IET reaction. A spontaneous flow of electrons from an organic to an aqueous redox couple requires $\Delta_{o}^{w}\varphi_{IET}^{0}<0\;\;V$ , such that ${\Delta_{o}^{w}G}_{IET}^{0}$ is negative [Equation (2)], while a spontaneous flow of electrons from an aqueous to an organic redox couple requires $\Delta_{o}^{w}\varphi_{IET}^{0}>0\;\;V$ , such that ${\Delta_{w}^{o}G}_{IET}^{0}$ is negative [Equation (3)]. Therefore, if $\Delta_{o}^{w}\varphi_{IET}^{0}$ >0 V, an external driving force, such as varying the applied $\Delta_{o}^{w}\varphi$ at the polarised L|L interface, would be required to drive a thermodynamically uphill IET reaction from an organic to an aqueous redox couple. However, a major conclusion of this article is that the latter is only possible *indirectly* under certain circumstances, as discussed *vide infra*.

Several predictions are possible from Figures 1a and b, prepared using the data summarized in Table S1. For the biphasic ORRs: (i) none of the ferrocene derivatives chosen for study are capable of spontaneously reducing O_2_ to HO_2_⋅ or O_2_⋅^−^ at any pH between 0.5 and 12, (ii) all of the ferrocene derivatives are capable of spontaneously reducing O_2_ via the 4e^−^ ORR to H_2_O, with only the weakest electron donor DiMFc being limited to pH≤ 9, and (iii) only DcMFc and PMFc are capable of spontaneously reducing O_2_ *via* the 2e^−^ ORR to H_2_O_2_, with DcMFc being limited to pH≤ 9 and PMFc being limited to pH≤ 3. The biphasic HERs are thermodynamically less favored than the biphasic ORRs with: (i) none of the ferrocene derivatives capable of spontaneously reducing aqueous protons (H_3_O^+^) to H_2_ and (ii) only DcMFc capable of spontaneously reducing interfacial [H^+^…TB^−^] to H_2_ and limited to pH≤ 6. In this case, *E* ^0^ of the interfacial [H^+^…TB^−^] in the mixed solvent region as a function of pH is taken as the average of *E* ^0^ of aqueous H_3_O^+^ at each pH and *E* ^0^ of H^+^ solubilised in TFT (Table S1).

Even if a biphasic single‐step IET reaction is predicted to be thermodynamically spontaneous, this does not necessarily mean it will be observed within the limits of the PPW at the polarised aqueous|TFT interface (approximately −0.4 to +0.6 V). If the difference between the standard redox potentials of the aqueous and organic redox couples is too large (typically >0.3 V), then single‐step IET may occur at an applied $\Delta_{o}^{w}\varphi$ outside the negative or positive limits of the PPW. To identify which biphasic single‐step IET reactions are predicted to appear within the PPW limits, $\Delta_{o}^{w}\varphi_{IET}^{0}$ for each biphasic O_2_ and proton reduction reaction with DcMFc and DiMFc were plotted as a function of pH in Figures 1c and d, respectively, using the data summarized in Tables S2 and S3. The corresponding plots with PMFc are shown in Figures S2a and b for clarity. Once more, from these figures, several predictions are possible. For the biphasic ORRs: (i) the 4e^−^ ORR with DcMFc at pH≤ 10, with PMFc at pH≤ 6, and with DiMFc at pH≤ 2 lies beyond the negative limits of the PPW and (ii) the 2e^−^ ORR with DcMFc at pH≥ 2, and PMFc and DiMFc at all pH values between 0.5 and 12 lies within the limits of the PPW. For the biphasic HERs: (i) the reduction of aqueous H_3_O^+^ with DcMFc at pH≥ 10, PMFc at pH≥ 6 and DiMFc at at pH≥ 2 lies beyond the positive limits of the PPW and (ii) the reduction of interfacial [H^+^…TB^−^] with all the ferrocene derivatives lies within the limits of the PPW at all pH values between 0.5 and 12.

In certain cases, even if a biphasic single‐step IET reaction is predicted to be thermodynamically spontaneous and lie within the PPW limits, the reaction may not be feasible for mechanistic reasons. DcMFc has previously been shown to facilitate the 2e^−^ ORR homogeneously in acidified organic electrolyte^[44]^ and biphasically at a polarised L|L interface^[22]^ via the mechanism described in Scheme 1a. However, to date, the only organic electron donor reported to achieve the biphasic 4e^−^ ORR is tetrathiafulvalene via a unique mechanism involving proton transfer to the organic phase (similar to the mechanism described in Scheme 1a) followed by the homogenous formation of stable helical tetramers in the organic phase from dimers between neutral and protonated tetrathiafulvalene molecules.^[45]^ In contrast, ferrocene derivatives are only capable of realising the biphasic 4e^−^ ORR in the presence of an interfacial molecular redox catalyst^[27]^ via the mechanism described in Scheme 1b. By themselves, DcMFc, PMFc and DiMFc lack the abilities to form the intermediate peroxo‐bridges required to enable the cleavage of the O−O bond and lead selectively to H_2_O and not H_2_O_2_ formation. Additionally, the dynamic nature of the L|L interface, as it is polarised, inhibits the biphasic 4e^−^ ORR in the absence of an interfacial molecular redox catalyst. At solid electrode|electrolyte interfaces, while the nature of the charge transfer is different for d‐ (Au, Pt, Pd) and π‐ (graphene) orbitals towards the ORR, the strong association between each solid substrate and the ORR intermediates yields molecular configurations that facilitate the cleavage of the O−O bond and dissociation of the ORR intermediates to selectively form H_2_O.^[46]^ However, at a polarised L|L interface, the association between ORR intermediates and the interface is weak due to a constant dynamic competition for interfacial protons between ORR intermediates and interactions to compensate the charge of TB^−^ (required to charge the interface to keep the desired applied $\Delta_{o}^{w}\varphi$ stable). Consequently, the ORR intermediates are not tightly bound to the L|L interface, leading to molecular configurations that do not enable the cleavage of the O−O bond, and thus are more likely to yield H_2_O_2_ as the ORR product.

To summarise, the only biphasic ORRs or HERs predicted to be thermodynamically spontaneous, mechanistically feasible and lie within the PPW limits are (i) the 2e^−^ ORR with DcMFc between pH 2 and 9 and PMFc at pH≤ 3 and (ii) the reduction of interfacial [H^+^…TB^−^] to H_2_ with DcMFc at pH≤ 6. In addition, the onset potentials of each of these biphasic IET reactions are predicted to be experimentally observed at negative applied $\Delta_{o}^{w}\varphi$ values.

### Experimentally probing the biphasic 2e^−^ ORR with a series of ferrocene‐derivatives

**Figure 2:**
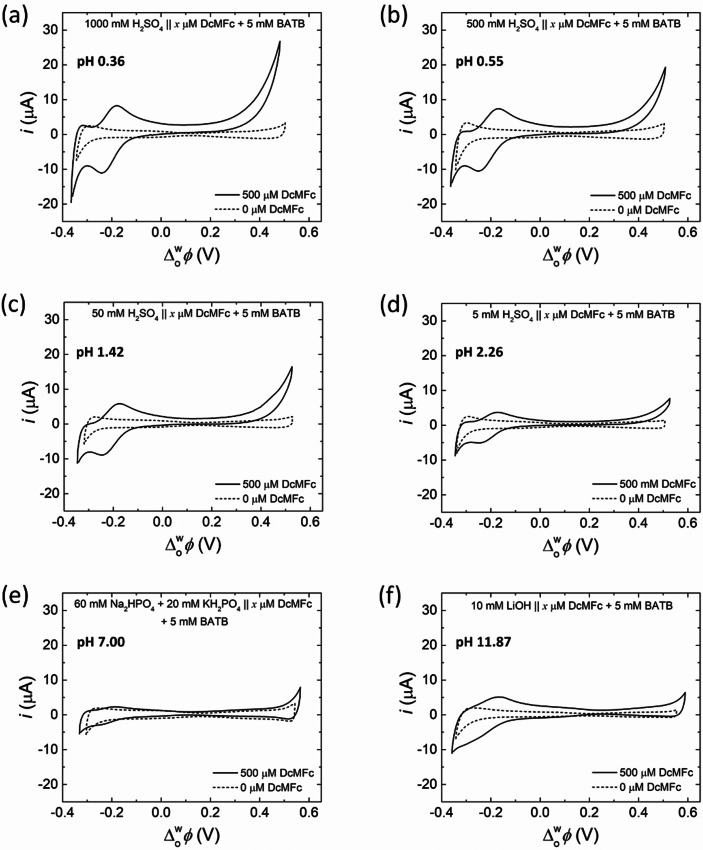
Experimental observations of the biphasic 2e^−^ ORR with DcMFc at a polarised L|L interface at different pHs. Cyclic voltammograms (CVs) were obtained in the presence (solid) and absence (dashed) of 500 μM DcMFc at pH (a) 0.36 (b) 0.55 (c) 1.42, (d) 2.26, (e) 7.00 and (f) 11.87. All CVs were obtained at a scan rate of 20 mV ⋅ s^−1^ using a shortened PPW that excluded the possibility of facilitated proton transfer to the organic phase by DcMFc at positive applied $\Delta_{o}^{w}\varphi$ . Experiments at pH 0.36, 0.55, 1.42 and 2.26 were carried out using Electrochemical Cell 1, at pH 7.00 using Electrochemical Cell 2 and at pH 11.87 using Electrochemical Cell 3 under aerobic, ambient conditions (see Scheme 2). The compositions of the aqueous and organic phases for each electrochemical cell are further noted in each panel.

**Scheme 2:**
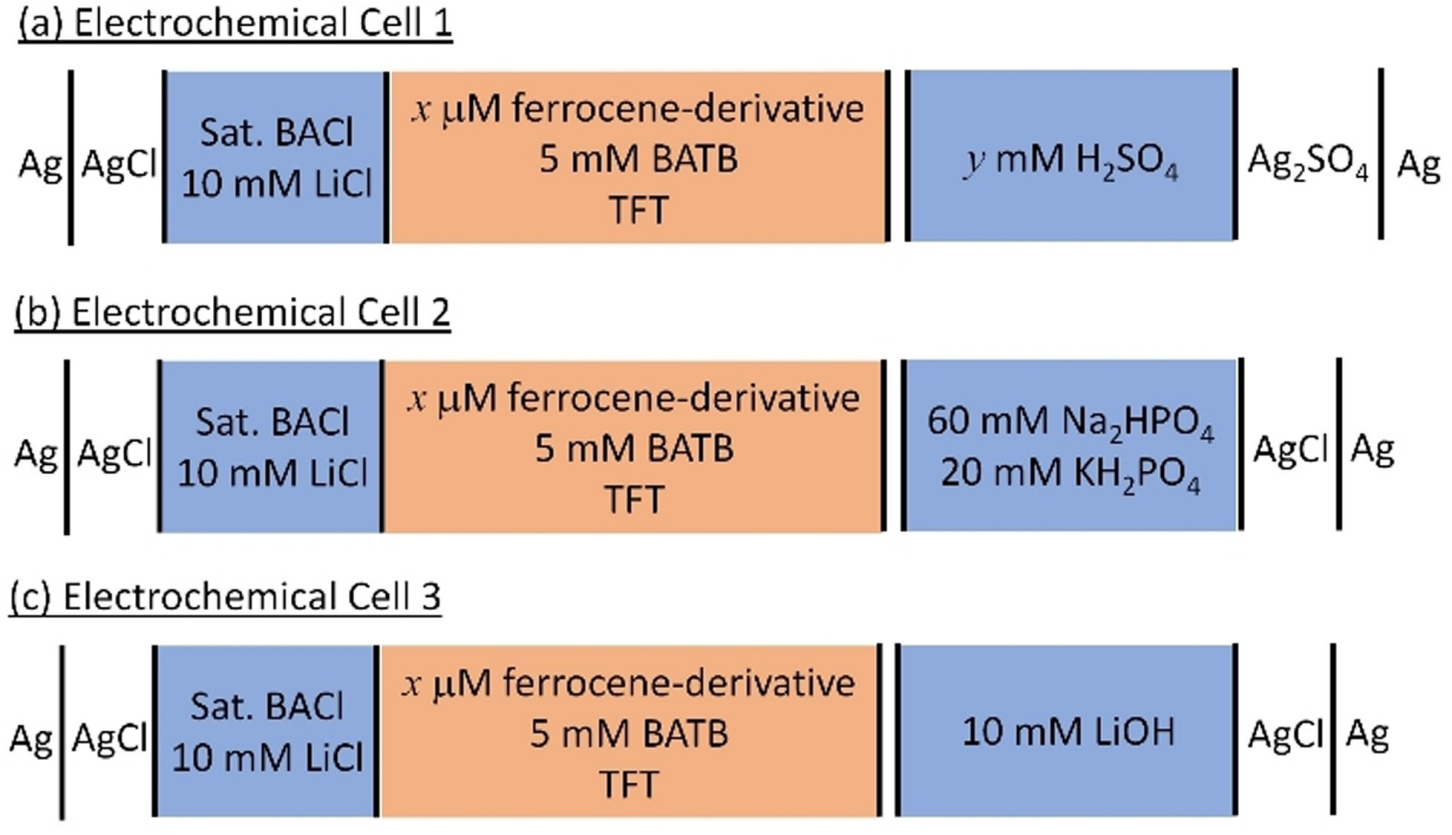
Electrochemical cell configurations of the four‐electrode electrochemical cells used. The ferrocene derivatives investigated were DcMFc, PMFc and DiMFc. In Electrochemical Cell 1, the H_2_SO_4_ concentration (*y* mM) was adjusted to vary the pH from 0.36 to 2.26. The concentration of ferrocene derivative in the TFT organic phase is *x* μM. For blank experiments in their absence *x*=0, and for experiments with a ferrocene derivative *x*=500 unless stated otherwise. In the organic reference solution, BACl is bis(triphenylphosphoranylidene)ammonium chloride, while BATB is the organic electrolyte salt bis(triphenylphosphoranylidene)ammonium tetrakis(pentafluorophenyl)borate. In this four‐electrode configuration, the Pt electrode in the organic phase and Ag/AgCl electrode in the organic reference solutions (saturated BACl and 10 mM LiCl) were connected to the counter and reference terminals, respectively, while the Pt and Ag/AgCl (or Ag/Ag_2_SO_4_) electrodes in the aqueous phase were connected to the working and sensing terminals, respectively. All electrochemical experiments were carried out under aerobic, ambient conditions.

To test the validity of the thermodynamic predictions in the previous section that identify which biphasic 2e^−^ ORRs may be observable at a polarised aqueous|TFT interface, cyclic voltammograms (CVs) were obtained in the presence and absence of DcMFc (Figure 2), PMFc (Figure S3) and DiMFc (Figure S4) at a selection of pH values between 0.36 and 11.87 under aerobic conditions. The four‐electrode electrochemical cell configurations at acidic, neutral, and basic conditions are described in Scheme 2.

For a series of pH <3 (pH 0.36, 0.55, 1.42 and 2.26), the 2e^−^ ORR with DcMFc was observed by cyclic voltammetry using a shortened PPW that excluded the possibility of facilitated ion transfer of aqueous protons by the metallocene species (Figures 2a to d). The CVs gave two distinct electrochemical signals compared to control CVs in the absence of DcMFc: (i) an irreversible rise in current at positive potentials, which increased in magnitude at more acidic conditions, and is attributed to the IET reaction involving O_2_, interfacial protons ([H^+^…TB^−^]) and DcMFc described in Scheme 1d, and (ii) a reversible ion transfer signal at −0.21 V, attributed to the reversible ion transfer of DcMFc^+^ produced during the biphasic IET reaction. At neutral (pH 7.00) and basic conditions (pH 11.87) with DcMFc (Figures 2e and f), no electrochemical signals due to IET were found. Furthermore, at acidic (pH 0.55), neutral (pH 7.00) and basic conditions (pH 11.87), with both PMFc (Figure S3) and DiMFc (Figure S4), no electrochemical signals due to IET were found. Interestingly, the oxidation of DcMFc seems to be enhanced at pH 11.87 independent of any biphasic IET, with a somewhat enhanced DcMFc^+^ ion transfer, but no IET signal, observed at this pH. The oxidation of DcMFc in alkaline pH may be due to a contaminant (a trace metallic ion) coming from the coating of the spatula due to reaction with LiOH upon introducing LiOH to the aqueous solution. The trace metallic ions will instantly be reduced by the DcMFc at the polarised L|L interface and therefore produce some DcMFc^+^ species. We believe that a trace contaminant may be present as the peak intensity of the DcMFc^+^ species produced is not constant, varying from 2 to 5 μ A between experiments.

**Figure 3:**
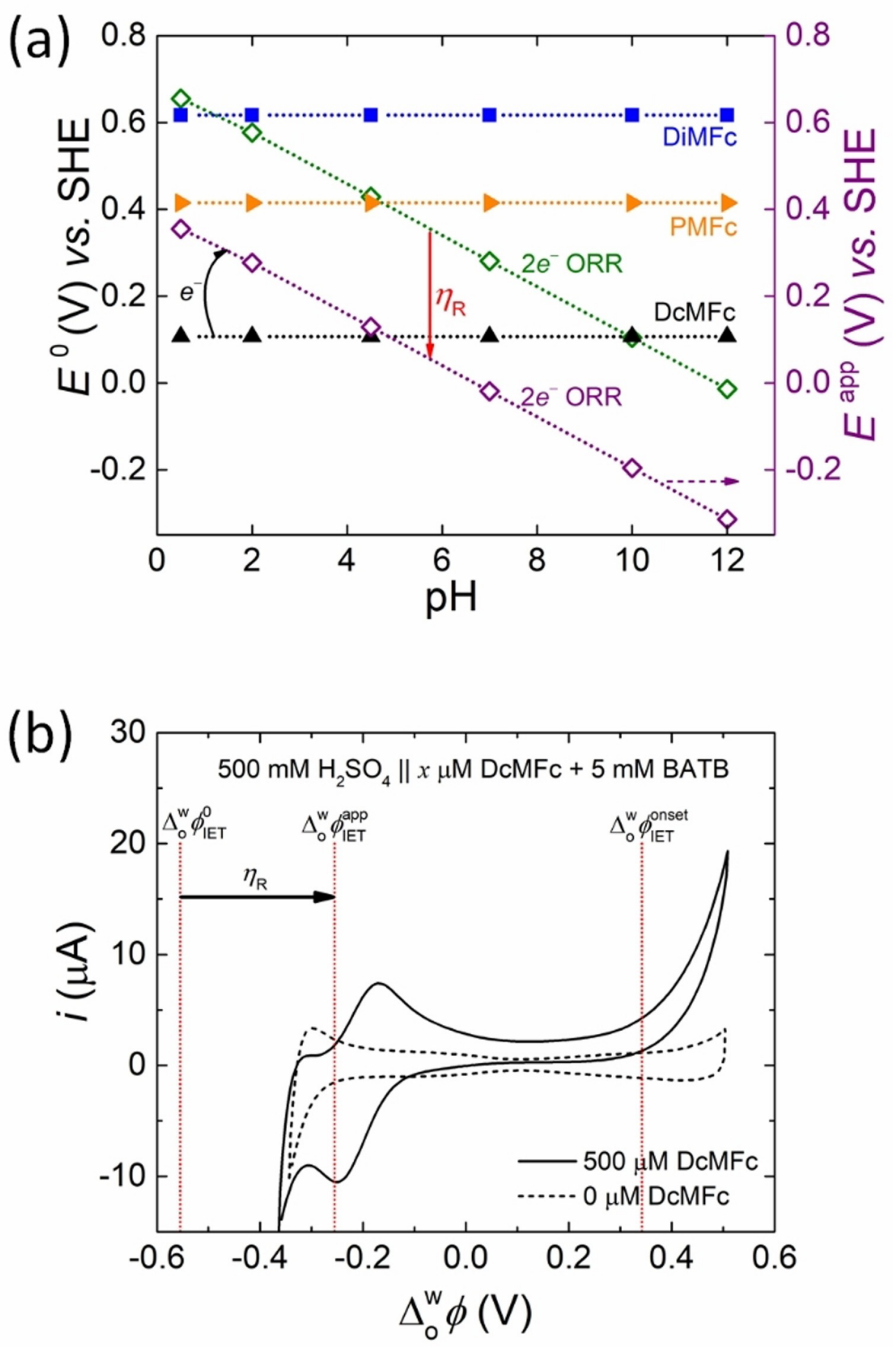
(a) Taking the intrinsic overpotential ($\eta_{R}$ ) of the biphasic 2e^−^ ORR at a polarised L|L interface into account, the apparent redox potential ($E^{app}$ , purple diamonds) required for O_2_ to undergo a biphasic 2e^−^ ORR shifts negatively compared with $E^{0}$ (green diamonds). (b) In turn, as overlaid on the CV obtained at pH 0.55 in the presence of 500 μM DcMFc at a scan rate of 20 mV s^−1^ (shown previously in Figure 2b), the apparent Galvani IET potential $(\Delta_{o}^{w}\varphi_{IET}^{app}$ ) shifts positively by $\eta_{R}$ on the Galvani scale compared with $\Delta_{o}^{w}\varphi_{IET}^{0}$ . The positive shift of $\Delta_{o}^{w}\varphi_{IET}^{app}$ is also shown in a plot of $\Delta_{o}^{w}\varphi$ *versus* pH for the biphasic 2e^−^ ORR with DcMFc in Figure S5. A further reason for the positive shift of $\Delta_{o}^{w}\varphi_{IET}^{app}$ compared to $\Delta_{o}^{w}\varphi_{IET}^{0}$ is that the $\Delta_{o}^{w}\varphi_{IET}^{0}$ values were calculated using the $E^{0}$ values for ORRs with aqueous H_3_O^+^ as the proton source, whereas more negative $E^{app}$ values for ORRs involving interfacial [H^+^…TB^−^] as the proton source may be more appropriate. Clearly, a major mismatch exists between the predicted value of $\Delta_{o}^{w}\varphi_{IET}^{app}$ and the experimentally observed onset interfacial Galvani potential difference ($\Delta_{o}^{w}\varphi_{IET}^{onset}$ ) for the biphasic 2e^−^ ORR with DcMFc, which appears at a much more positive applied $\Delta_{o}^{w}\varphi$ .

While these experimental data are broadly in line with the overall thermodynamic predictions, the 2e^−^ ORR was electrochemically observed over a narrower and more acidic pH range (between pH 0.36 and 2.26) than predicted (between pH 2 and 9) with DcMFc and no evidence of the 2e^−^ ORR was found with PMFc at pH≤ 3. To explain these discrepancies, the concept of an intrinsic overpotential ($\eta_{R}$ ) of the biphasic 2e^−^ ORR at a polarised L|L interface is considered. The origin and magnitude of $\eta_{R}$ will differ for each biphasic single‐step IET reaction due to factors such as reorganisation energies and double‐layer effects.[^37^, ^43^] The apparent redox potential ($E^{app}=E^{0}-\eta_{R}$ ) required for O_2_ to undergo a biphasic 2e^−^ ORR shifts negatively vs. SHE with increasing $\eta_{R}$ compared to $E^{0}$ (Figure 3a), while the apparent Galvani IET potential $\left(\Delta_{o}^{w}\varphi_{IET}^{app}=\Delta_{o}^{w}\varphi_{IET}^{0}+\eta_{R}\right)$ shifts positively on the Galvani scale compared to $\Delta_{o}^{w}\varphi_{IET}^{0}$ (Figure S5). Setting $\eta_{R}=+0.3\;\;V$ fully addresses the discrepancies between the experimental data in Figures 2, S3 and S4 and the thermodynamic predictions with (i) *E* ^0^ of PMFc now being more positive than $E^{app}$ of the 2e^−^ ORR, meaning that IET is no longer predicted to proceed spontaneously at any pH between 0.5 and 12, and (ii) the 2e^−^ ORR with DcMFc is now predicted to be spontaneous only at pH≤ 3.

Furthermore, in addition to the presence of an intrinsic overpotential to biphasic single‐step IET reactions at the polarised L|L interface, the discrepancies may be due to the $\Delta_{o}^{w}\varphi_{IET}^{0}$ values for the various biphasic ORRs in Figure 1c being calculated using the $E^{0}$ values for ORRs involving O_2_ and bulk aqueous H_3_O^+^ (as plotted in Figure 1a). However, as is the case for the biphasic HERs described in Figures 1b and d, the true source of protons for the biphasic ORRs are interfacial [H^+^…TB^−^]. Thus, $E^{app}$ shifts negatively vs. SHE for ORRs involving interfacial [H^+^…TB^−^] compared to $E^{0}$ for ORRs involving aqueous H_3_O^+^, and therefore $\Delta_{o}^{w}\varphi_{IET}^{app}$ for the various biphasic ORRs shifts positively on the Galvani scale compared to $\Delta_{o}^{w}\varphi_{IET}^{0}$ . This point, that there may be substantial differences between the $E^{0}$ value determined for a redox couple in the bulk aqueous or organic phase and the $E^{app}$ value for that redox couple in the mixed solvent region, will be explored in greater detail *vide infra*.

From the literature, the biphasic reduction of protons to H_2_ by DcMFc has only been observed at pH≤ 3,[^47^, ^48^, ^49^] and not at the more extended pH range of ≤ 6 predicted by our purely thermodynamic analysis. In this case, the discrepancies between the experimental data in the literature and the thermodynamic predictions were resolved by setting $\eta_{R}=+0.12\;\;V$ (Figure S6), suggesting that $\eta_{R}$ is more substantial for O_2_ reduction than proton reduction at a polarised L|L interface.

A conclusion from this section is that biphasic single‐step IET reactions that are mechanistically feasible at a polarised L|L interface, such as the 2e^−^ ORR, and are predicted to be spontaneous, even after the consideration of an intrinsic overpotential to the biphasic single‐step IET as described by Equation (4), may lead to an observable IET signal within the PPW at the polarised L|L interface.

(4) $$\Delta_{o}^{w}\varphi_{IET}^{app}={\left[E_{D^{+}/D}^{0}\right]}_{SHE}^{TFT}-\left({\left[E_{A/A^{-}}^{0}\right]}_{SHE}^{aq}-\eta_{R}\right)<0\;\;V.$$

Furthermore, in our analysis, any biphasic single‐step IET reactions with $\Delta_{o}^{w}\varphi_{IET}^{app}>0\;\;V$ involving electron transfer from an organic to aqueous redox couple gave no observable IET signal within the PPW. This supports the hypothesis that the applied $\Delta_{o}^{w}\varphi$ provides no direct driving force to realise a thermodynamically uphill reaction, contrary to classical electron transfer theory at solid electrode|electrolyte interfaces.

As noted, for the biphasic 2e^−^ ORR with DcMFc in Figure 2, only those pH values that lead to biphasic single‐step IET reactions with $\Delta_{o}^{w}\varphi_{IET}^{app}<0\;\;V$ experimentally gave signals due to IET. Thus, by default, each of these reactions were expected to be seen at negative $\Delta_{o}^{w}\varphi$ values. However, this was not the case, with the onset potential for the biphasic single‐step IET reaction ($\Delta_{o}^{w}\varphi_{IET}^{onset}$ ) always observed at positive $\Delta_{o}^{w}\varphi$ values that were much more positive than $\Delta_{o}^{w}\varphi_{IET}^{0}$ or $\Delta_{o}^{w}\varphi_{IET}^{app}$ . To highlight this discrepancy, dotted lines corresponding to $\Delta_{o}^{w}\varphi_{IET}^{0}$ , $\Delta_{o}^{w}\varphi_{IET}^{app}$ and $\Delta_{o}^{w}\varphi_{IET}^{onset}$ are overlaid on the CV obtained at pH 0.55 in the presence of 500 μM DcMFc, see Figure 3b. While the applied $\Delta_{o}^{w}\varphi$ provides no driving force to realise a thermodynamically uphill reaction, it does substantially influence the nature of the ions present on either side of the L|L interface (i. e., aqueous cations or anions, organic cations or anions) and, in particular, their interfacial concentrations. In the following section, the PZC at the polarised L|L interface is discussed and its relationship with $\Delta_{o}^{w}\varphi_{IET}^{onset}$ for a spontaneous biphasic single‐step IET reaction outlined.

### Correlation of the onset potential for the biphasic 2e^−^ ORR with DcMFc and the PZC

**Figure 4:**
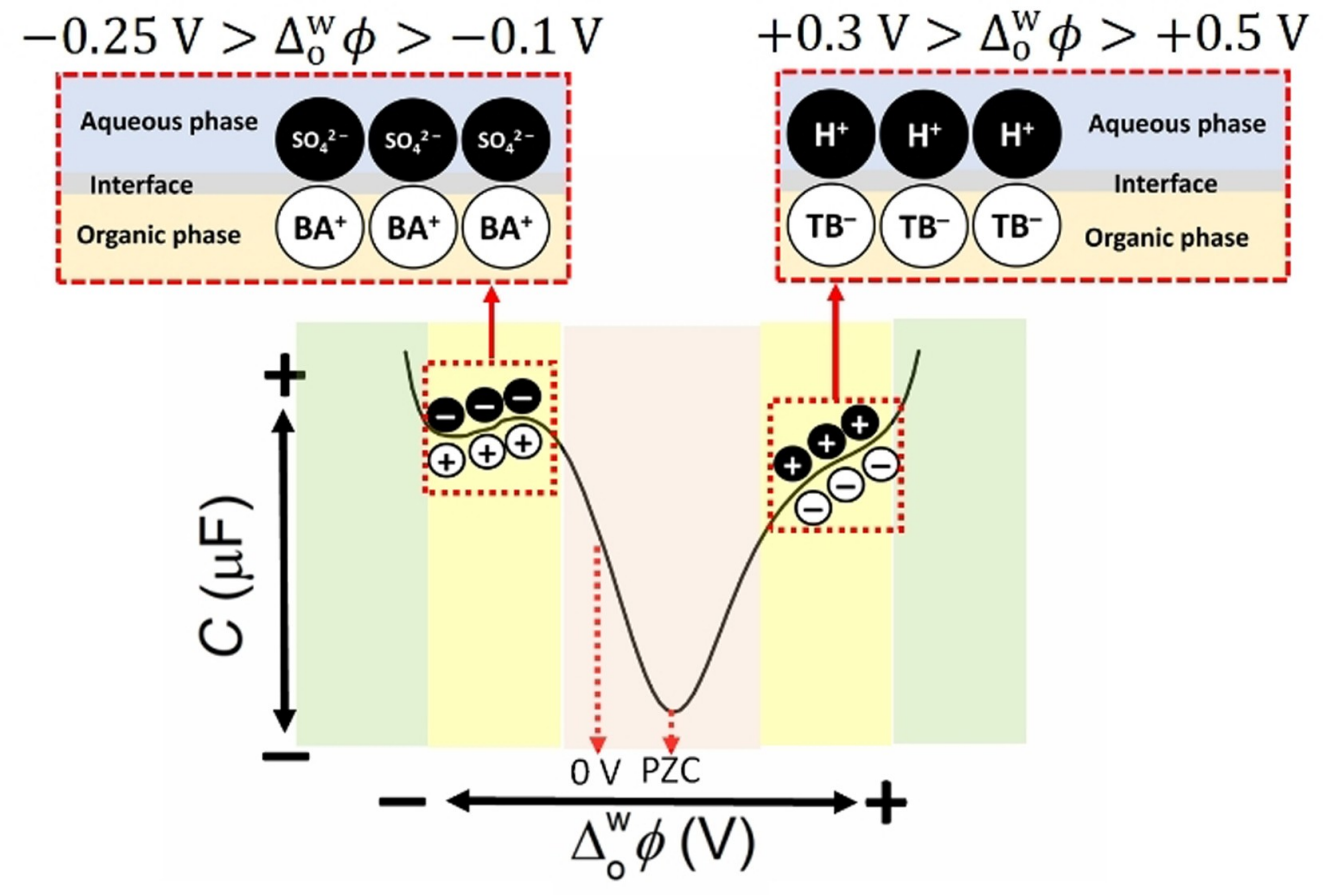
Differential capacitance measurements highlight three distinct regions of ion adsorption and ion transfer at a polarised L|L interface. (i) For the electrochemical cell investigated, the potential of zero charge (PZC) is located at +0.13 V in the orange shaded area. (ii) At potentials approximately 200 to 300 mV positive and negative of the PZC, distinct plateaus in the differential capacitance appear (yellow shaded areas). For applied $\Delta_{o}^{w}\varphi$ values between +0.3 and +0.5 V, the EDL on the organic side of the L|L interface is saturated with TB^−^ and the interfacial [H^+^…TB^−^] concentration plateaus. For applied $\Delta_{o}^{w}\varphi$ values between −0.1 and −0.25 V, the EDL on the organic side of the L|L interface is saturated with BA^+^ and the interfacial [SO_4_^2−^…BA^+^] concentration plateaus. (iii) Potentials positive of +0.5 V and negative of −0.25 V (green shaded areas) induce faradaic ion transfer of aqueous cations (protons) and anions (SO_4_^2−^), respectively, to the organic phase. The differential capacitance measurement (see Figure S7 for the raw measurement) was taken with a voltage excitation frequency of 5 Hz using Electrochemical Cell 1 (see Scheme 2a) under aerobic, ambient conditions.

The PZC represents a “switching point” in the nature of the ionic distribution within the back‐to‐back EDLs at a polarised L|L interface. Differential capacitance measurements provide a vivid description of how the ionic distribution changes at potentials positive and negative of the PZC (Figure 4),^[16]^ and the raw differential capacitance measurement used to prepare the scheme is shown in Figure S7. The PZC itself represents the applied $\Delta_{o}^{w}\varphi \;$ where the minimum interfacial capacitance is recorded with the lowest concentration of aqueous and organic ions present at the L|L interface (ca. +0.13 V in Figure 4). At potentials positive of the PZC, the EDL on the aqueous side of the L|L interface experiences a build‐up of positive charge (due to accumulation of cations and loss of anions), and the EDL on the organic side experiences a build‐up of negative charge (due to the accumulation of anions primarily, as modelled *vide infra*). The reverse processes occur at potentials negative of the PZC.

For the electrochemical configuration of the biphasic 2e^−^ ORR with DcMFc system at acidic conditions described in Scheme 2a, the interfacial concentrations of aqueous protons and organic TB^−^ anions, and thus the interfacial capacitance, begin to increase at potentials positive of the PZC (orange shaded area positive of the PZC in Figure 4). At a certain applied $\Delta_{o}^{w}\varphi$ positive of the PZC (ca. +0.37 V), the EDL on the organic side of the L|L interface is saturated with TB^−^ and the interfacial [H^+^…TB^−^] concentration, and thus interfacial capacitance, plateaus (yellow shaded area positive of the PZC in Figure 4). As the applied $\Delta_{o}^{w}\varphi \;$ is scanned progressively more positive (> +0.5 V), ion transfer of aqueous cations to the organic phase and/or organic anions to the aqueous phase takes place (green shaded area positive of the PZC in Figure 4). In this region, the width of mixed solvent region increases and the L|L interface begins to emulsify, leading to substantial changes in the interfacial tension, viscosity and relative permittivity. Similar trends are observed scanning to potentials negative of the PZC, with the EDL on the organic side of the L|L interface being saturated with BA^+^ that is compensated by sulfate (SO_4_^2−^) and bisulfate (HSO_4_^−^). For clarity, only the interfacial [SO_4_^2−^…BA^+^] interaction is depicted in Figure 4.

For the biphasic 2e^−^ ORR with DcMFc to proceed, three species must meet simultaneously at the aqueous|TFT interface: O_2_, protons and DcMFc. Both O_2_ and DcMFc are neutral species, with O_2_ dissolved in both the aqueous and TFT phases and DcMFc only dissolved in the TFT phase. Thus, the applied $\Delta_{o}^{w}\varphi \;$ does not influence their interfacial concentrations. However, as shown in Figure 4, the concentration of interfacial [H^+^…TB^−^], reaches a maximum saturation between ca. +0.30 and +0.50 V. This narrow potential range, where interfacial protons are plentiful, coincides precisely with the potential range where the biphasic 2e^−^ ORR with DcMFc is observed at pH≤ 3 in Figures 2a‐d. All previous biphasic 2e^−^ ORR studies with a ferrocene derivative directly involved aqueous H_3_O^+^ either (i) undergoing facilitated ion transfer to the organic phase assisted by a ferrocene derivative (Scheme 1a), (ii) undergoing a PCET reaction (Scheme 1b) or (iii) being involved in a bipolar mechanism (Scheme 1c), with an associated ca. 59 mV/pH shift of the onset potential of the irreversible rise in current indicative of the biphasic ORR reaction in each case. However, the mechanism described herein is distinct as it involves [H^+^…TB^−^] as the interfacial proton source (Scheme 1d) and, thus, there is no expectation that $\Delta_{o}^{w}\varphi_{IET}^{onset}$ will shift ca. 59 mV/pH shift for experiments systematically varying the bulk aqueous pH.

**Figure 5:**
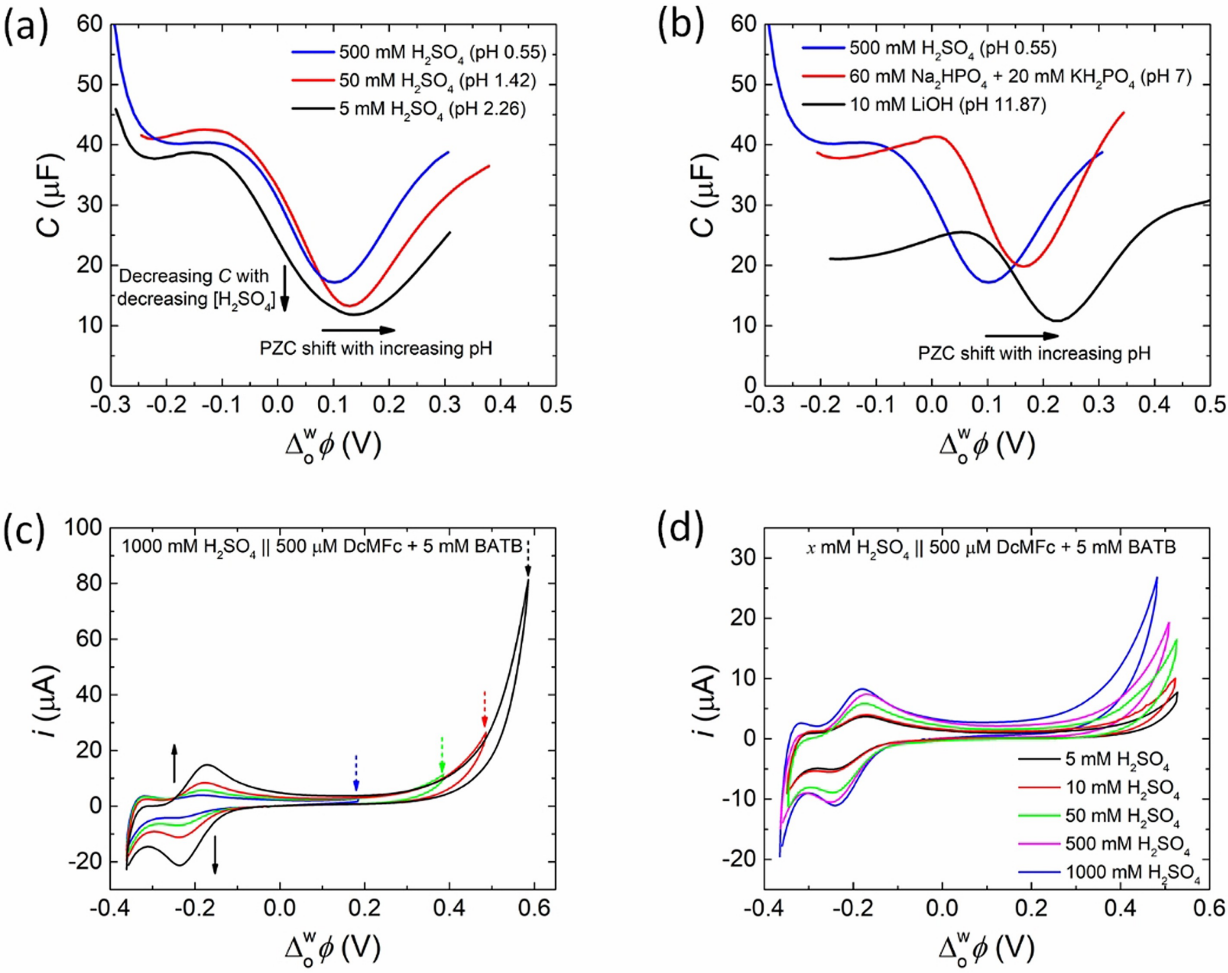
(a, b) Differential capacitance measurements demonstrate that the PZC shifts positively with the pH of the aqueous phase from pH 0.55 to 11.87. (c) Extending the PPW to progressively more positive potentials led to a large increase in current after +0.5 V. At applied $\Delta_{o}^{w}\varphi$ values positive of +0.5 V, the mechanism of biphasic O_2_ reduction shifts from one based on single‐step IET involving interfacial protons ([H^+^…TB^−^]), as proposed to occur between +0.3 and +0.5 V in Scheme 1d, to multi‐step proton transfer followed by a homogeneous ORR, as depicted in Scheme 1a. (d) CVs obtained in the presence of 500 μM DcMFc with increasing H_2_SO_4_ concentrations from 5 to 1000 mM. The positive end of the PPW was restricted to +0.5 V to avoid facilitated ion transfer of protons to the organic phase. Differential capacitance measurements in (a, b) were taken using a voltage excitation frequency of 5 Hz at pH 0.55, 1.42 and 2.26 using Electrochemical Cell 1, at pH 7.00 using Electrochemical Cell 2 and at pH 11.87 using Electrochemical Cell 3 under aerobic, ambient conditions (see Scheme 2). All CVs in (c, d) were carried out at a scan rate of 20 mV s^−1^ using Electrochemical Cell 1 under aerobic, ambient conditions.

To conclusively correlate the relationship between the PZC and $\Delta_{o}^{w}\varphi_{IET}^{onset}$ for a spontaneous biphasic 2e^−^ ORR with DcMFc, the changes of both the PZC and $\Delta_{o}^{w}\varphi_{IET}^{onset}$ with pH were determined and compared. Differential capacitance measurements demonstrate that the PZC shifts positively with the pH of the aqueous phase from pH 0.55 to 11.87 with a shallow slope of ca. 10 mV/pH (Figures 5a and b). Control differential capacitance measurements demonstrate that the PZC does not shift meaningfully in the presence of a neutral electron donor, in this case 500 μM PMFc, from pH 0.55 to 11.87 (Figure S8). Once more, as was the case in Figure 2, to ensure biphasic single‐step IET took place between DcMFc and [H^+^…TB^−^], and the mechanism initiated by ion transfer of aqueous H_3_O^+^ to the organic phase in Scheme 1a was avoided, the positive‐edge of the PPW was carefully limited. Differential capacitance measurements are more sensitive to determining the PPW and detecting background faradaic currents than cyclic voltammetry. Thus, based on the differential capacitance measurement in Figure S7, the positive switching potential was set to +0.5 V. This decision was validated by recording CVs with a progressively increased positive switching potential from +0.2 to +0.6 V, see Figure 5c. Clearly, a rapid increase in current is observed after +0.5 V due to significant ion transfer of aqueous H_3_O^+^ to the organic phase, with an associated increase in the magnitude of the current peaks at negative potentials due to the reversible ion transfer of DcMFc^+^. CVs were subsequently recorded for a series of H_2_SO_4_ concentrations (Figure 5d), with the pH ranging from 0.55 to 2.26, and the $\Delta_{o}^{w}\varphi_{IET}^{onset}$ determined.

**Figure 6:**
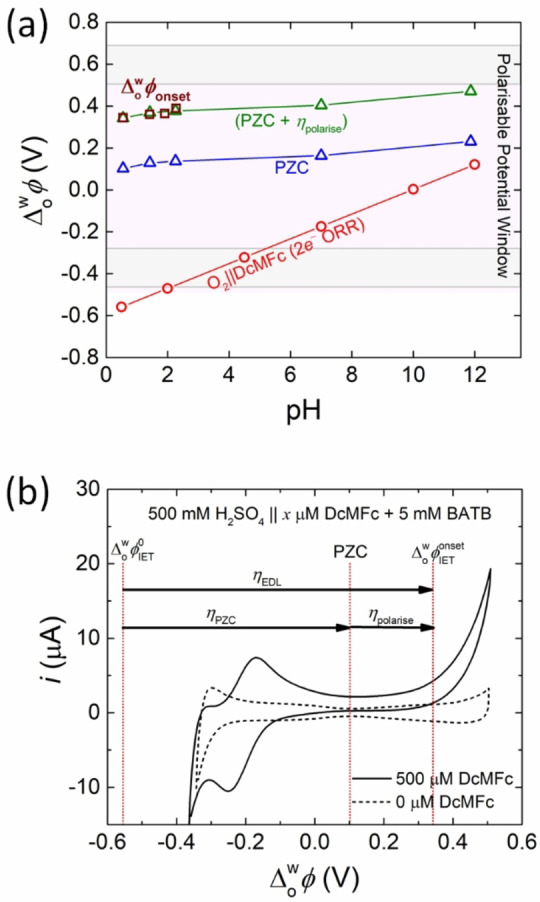
(a) A plot of $\Delta_{o}^{w}\varphi$ versus pH comparing the thermodynamically predicted $\Delta_{o}^{w}\varphi_{IET}^{0}\;$ values as a function of pH for the biphasic 2e^−^ ORR with DcMFc as the organic electron donor (red circles, determined as described in Figure 1c), the PZC as a function of pH (blue triangles, determined from the differential capacitance measurements shown in Figure 6a and b) and the $\Delta_{o}^{w}\varphi_{IET}^{onset}$ values for the biphasic 2e^−^ ORR with DcMFc at acidic pH (purple squares, determined from the CVs shown in Figure 6d). The overpotential required to polarise the L|L interface sufficiently positive of the PZC to generate the [H^+^…TB^−^] plateau region (as described in Figure 4) is assigned the value $\eta_{polarise}=+0.240\;\;V$ . The values of (PZC+$\eta_{polarise}$ ) (green triangles) overlap precisely with the $\Delta_{o}^{w}\varphi_{IET}^{onset}\;$ values at acidic pH. (b) As overlaid on the CV obtained at pH 0.55 in the presence of 500 μM DcMFc at a scan rate of 20 mV ⋅ s^−1^ (see Figure 2c), the overpotential to modulate the ion distribution in the back‐to‐back EDLs ($\eta_{EDL}$ ) to enable single‐step IET at each pH for the biphasic 2e^−^ ORR with DcMFc, and thus $\Delta_{o}^{w}\varphi_{IET}^{onset}$ , is determined by an overpotential to reach the PZC ($\eta_{PZC}$ ) plus $\eta_{polarise}$ .

The values of the thermodynamically predicted $\Delta_{o}^{w}\varphi_{IET}^{0}$ and experimentally determined $\Delta_{o}^{w}\varphi_{IET}^{onset}$ for the biphasic 2e^−^ ORR with DcMFc, and the PZC, are plotted versus pH in Figure 6a. A value of +0.240 V is assigned to the overpotential required to polarise the L|L interface ($\eta_{polarise}$ ) sufficiently positive of the PZC to generate the [H^+^…TB^−^] plateau region and a (PZC + $\eta_{polarise}$ ) value is also plotted versus pH. The value of $\eta_{polarise}$ was determined from the differential capacitance curve in Figure S7. Although, the pH range where ${\Delta_{o}^{w}\varphi }_{onset}$ could be measured is limited to pH≤ 3, the shift of $\Delta_{o}^{w}\varphi_{IET}^{onset}$ presents an identical slope to the shift of the PZC in this acidic pH range. Furthermore, the (PZC + $\eta_{polarise}$ ) values overlap precisely with the $\Delta_{o}^{w}\varphi_{IET}^{onset}$ values at acidic pH. Thus, the conclusion is that for the biphasic 2e^−^ ORR with DcMFc under thermodynamically spontaneous conditions at pH≤ 3 (with $\Delta_{o}^{w}\varphi_{IET}^{app}<0\;\;V\;$ ), biphasic single‐step IET between DcMFc, O_2_ and interfacial protons is kinetically inhibited until the applied $\Delta_{o}^{w}\varphi$ is sufficiently positive to modulate the ion distribution at the polarised L|L interface such that the interface is saturated with [H^+^…TB^−^]. Therefore, the overpotential to modulate the ion distribution in the back‐to‐back EDLs ($\eta_{EDL}$ ) to enable IET at each pH, and thus $\Delta_{o}^{w}\varphi_{IET}^{onset}$ , is determined by an overpotential to reach the PZC ($\eta_{PZC}$ ) plus $\eta_{polarise}$ as described by Equation 5:

(5) $$\Delta_{o}^{w}\varphi_{IET}^{onset}=\Delta_{o}^{w}\varphi_{IET}^{0}+\left(\eta_{PZC}+\eta_{polarise}\right)=\Delta_{o}^{w}\varphi_{IET}^{0}+\eta_{EDL}\;.$$

This conclusion is clearly illustrated by overlaying dotted lines corresponding to $\Delta_{o}^{w}\varphi_{IET}^{0}$ , the PZC and $\Delta_{o}^{w}\varphi_{IET}^{onset}$ on the CV obtained at pH 0.55 in the presence of 500 μM DcMFc, see Figure 6b, with solid arrows indicating $\eta_{EDL}$ , $\eta_{PZC}$ and $\eta_{polarise}$ . In the following section, the magnitude of the increase of the interfacial proton concentration upon saturation of the L|L interface with [H^+^…TB^−^] at an applied $\Delta_{o}^{w}\varphi$ positive of the PZC is demonstrated using a Verwey‐Niessen model of the polarised L|L interface.

### Modelling the increase in interfacial concentrations of ions in the EDLs on either side of the polarised L|L interface relative to the PZC as a function of the applied $\Delta_{o}^{w}\varphi$

**Figure 7:**
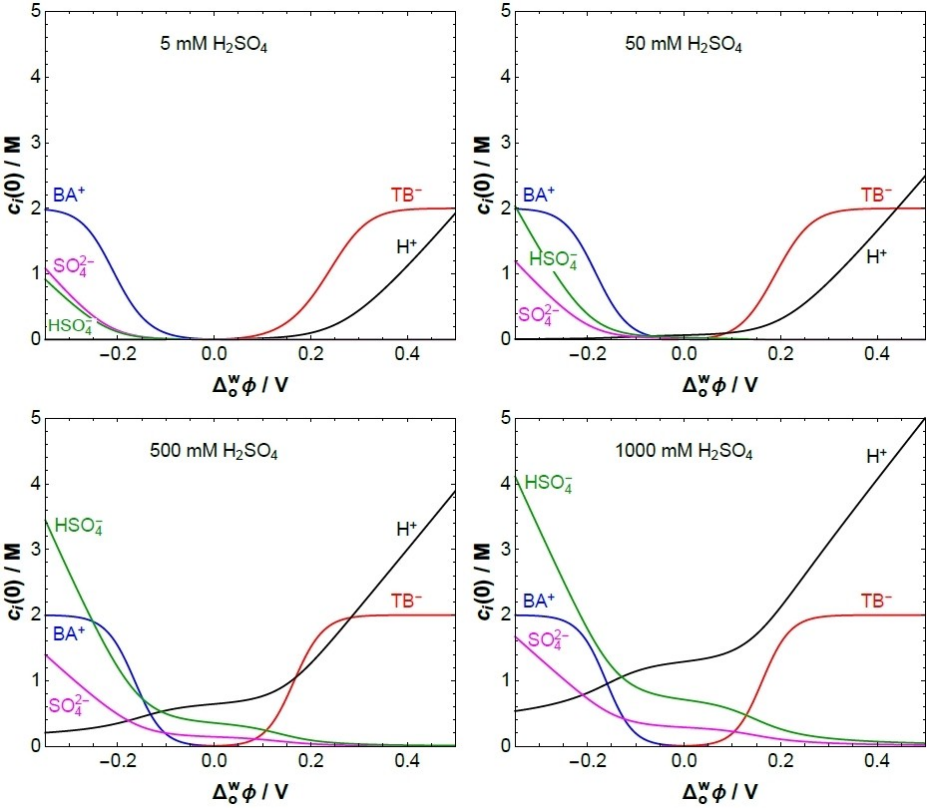
Interfacial concentrations vs. the applied interfacial Galvani potential difference $\Delta_{o}^{w}\varphi$ in Electrochemical Cell 1 (see Scheme 2a), 5 mM BATB (TFT)||*y* mM H_2_SO_4_ (aq), for *y*=5, 50, 500, and 1000 using a modified Verwey‐Niessen model that accounts for the finite size of the organic phase ions. Their maximum concentration has been given the roughly estimated value of 2 M. At the positive end of the PPW, the proton concentration takes very similar values for *y*=5, 50, 500, and 1000; just like the sulfate ion and bisulfate ion concentration in the negative end of the PPW.

**Figure 8:**
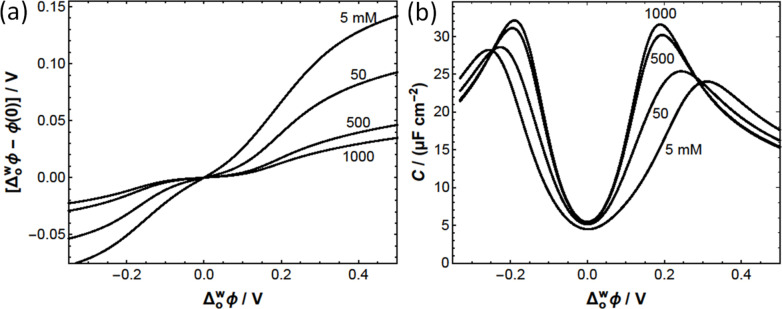
(a) The Galvani potential drop $\Delta_{o}^{w}\varphi -\varphi \left(0\right)$ in the aqueous phase, where $\varphi \left(0\right)$ is the potential at the L|L interface, is significantly smaller than $\Delta_{o}^{w}\varphi$ because most of the applied interfacial Galvani potential difference drops in the organic phase. These calculations correspond to Electrochemical Cell 1 (see Scheme 2a), 5 mM BATB (TFT)||*y* mM H_2_SO_4_ (aq), for *y*=5, 50, 500, and 1000, using a modified Verwey‐Niessen model that accounts for the finite size of the organic phase ions. Their maximum concentration has been given the roughly estimated value of 2 M. (b) The differential capacitance curves show local maxima due to finite ion size effects. The local maxima of the capacitance curves roughly correspond to the applied $\Delta_{o}^{w}\varphi$ where the organic ions reach their maximum concentration (see concentration profiles in Figure S10). The curvature of the capacitance curve at its minimum decreases with decreasing sulfuric acid concentration, in agreement with observations in Figure 5a.

The interfacial concentrations of ions can be calculated using a Verwey‐Niessen model of the polarised L|L interface, under the idealised situation of a flat interface with no solvent mixing (Figure 7). Most of the applied $\Delta_{o}^{w}\varphi$ drops on the organic side of the back‐to‐back EDLs. The Galvani potential drop $\Delta_{o}^{w}\varphi -\varphi \left(0\right)$ in the aqueous phase is significantly smaller than $\Delta_{o}^{w}\varphi /2$ , where $\varphi \left(0\right)$ is the potential at the L|L interface (Figure 8a). This is due to several factors. First, the relative electrical permittivity of TFT is significantly lower than that of water, which makes the interfacial electric field larger on the organic side than on the aqueous side.^[16]^ Second, the electrolyte concentration is typically larger in the aqueous phase than in the organic phase, so that the Debye screening is more effective in the former. The Galvani potential drop in the aqueous phase decreases with increasing electrolyte concentration in this phase. Third, and also quite relevant, the organic ions have a larger size than the aqueous ions, so that a modified Verwey‐Niessen model that accounts for finite ion size effects has to be used to calculate the interfacial concentrations.[^50^, ^51^, ^52^] This modified model also predicts that the potential drop in the EDL on the organic side of the L|L interface continues to increase with increasing $\Delta_{o}^{w}\varphi$ even after the interfacial concentrations of the organic ions have reached their maximum value.

The L|L interface is assumed to be ideally polarisable and with no specific ion adsorption, i. e., there is no EDL and no charge separation in the absence of applied potential, $\Delta_{o}^{w}\varphi =0$ , so that the PZC is zero in this model. As $\Delta_{o}^{w}\varphi$ is increased, the charge separated across the L|L interface increases. Although the interfacial concentrations of the organic and aqueous ions can be quite different, the EDL is globally electroneutral. The charge density $Q^{w}$ on the aqueous side of the EDL has the same sign as $\Delta_{o}^{w}\varphi$ and is exactly compensated by the charge density $Q^{o}$ on the organic side of the EDL, i. e., $Q^{w}=-Q^{o}$ . The contribution $Q_{i}$ of each ionic species in Electrochemical Cell 1 (Scheme 2a) to the separated charge density across the L|L interface is determined as a function of the applied $\Delta_{o}^{w}\varphi$ in Figure S9a. Note that $Q^{w}=Q_{H^{+}}+Q_{{HSO}_{4}^{-}}+Q_{{SO}_{4}^{2-}}=$ $-Q_{{BA}^{+}}-Q_{{TB}^{-}}=-Q^{o}$ . The differential capacitance is then calculated as ${C=dQ}^{w}/d\Delta_{o}^{w}\varphi$ , see Figures 8b and S9b. The rate of increase of the separated charge with the applied $\Delta_{o}^{w}\varphi$ reaches a maximum value approximately when the organic ions reach their maximum concentration at the L|L interface. A further increase in $\Delta_{o}^{w}\varphi$ can only lead to an increase of $Q^{w}=-Q^{o}$ by increasing the thickness of the EDL on the organic side, which results in a lower value of the differential capacitance. Thus, the capacitance curves show local maxima due to finite ion size effects (Figure 8b).^[53]^

The charge density $Q^{o}\;$ is mainly due to the accumulation of organic ions close to the interface, TB^−^ anions when $\Delta_{o}^{w}\varphi >0$ and BA^+^ cations when $\Delta_{o}^{w}\varphi <0$ ; because the bulk concentration of organic ions is so small that ion accumulation is the only mechanism to build up a significant $Q^{o}$ . On the contrary, when $\Delta_{o}^{w}\varphi >0\;$ the charge density $Q^{w}$ on the aqueous side of the EDL is due to both the accumulation of protons close to the interface and the depletion of SO_4_^2−^ and HSO_4_^−^. Since the Galvani potential drop on the aqueous side of the EDL is relatively small, protons are not completely depleted from the interfacial region even at the negative end of the PPW.

A key message from this section is that for biphasic systems comprising an aqueous solution with a millimolar acid electrolyte concentration and an organic solution containing a millimolar BATB electrolyte concentration, upon polarisation of the L|L interface to an applied $\Delta_{o}^{w}\varphi$ sufficiently positive of the PZC, molar concentrations of protons accumulate in the EDL on the aqueous side of the L|L interface to compensate the build‐up of molar concentrations of TB^−^ in the EDL on the organic side of the L|L interface. This substantial accumulation of interfacial [H^+^…TB^−^] protons, ultimately limited by the accumulation of organic TB^−^, accelerates the kinetics of the biphasic ORR with DcMFc and determines the value of $\Delta_{o}^{w}\varphi_{IET}^{onset}$ experimentally observed.

### Influence of the interfacial ion distributions in the EDLs either side of the polarised L|L interface on biphasic single‐step IET reactions: a general discussion

Our experimental data in Figures 2, S3 and S4 for the biphasic 2e^−^ ORR with DcMFc, PMFc and DiMFc as a function of pH revealed that only thermodynamically spontaneous biphasic single‐step IET reactions with $\Delta_{o}^{w}\varphi_{IET}^{app}<0\;\;V$ gave an observable IET signal within the PPW. This observation was specific for a biphasic system where electrons flow across the L|L interface from an organic to an aqueous redox couple. Therefore, a valid expectation is that for a biphasic system where electrons flow in the opposite direction, from an aqueous to an organic redox couple, only thermodynamically spontaneous biphasic single‐step IET reactions with $\Delta_{o}^{w}\varphi_{IET}^{app}>0\;\;V$ will give an observable IET signal within the PPW. Also, as shown in Figures 5a and b and Figure 6a, the PZC shifts slightly positive (from +0.10 to +0.23 V) with pH. Indeed, for the vast majority of biphasic systems, the PZC will not be found at 0 V, but at a value positive or negative of the PZC due to accumulation of ions on each side of the L|L interface.

**Figure 9:**
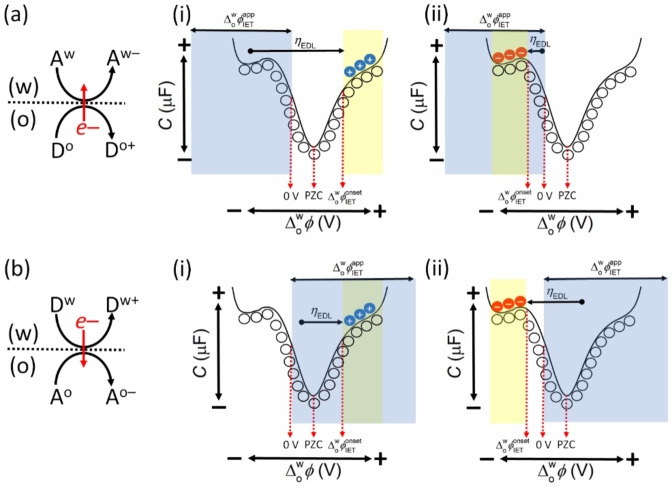
The distributions of the redox species on either side of the L|L interface as a function of the applied $\Delta_{o}^{w}\varphi$ are depicted by overlaying cationic (blue circle), anionic (red circle) or neutral (hollow circle) symbols on a differential capacitance measurement with features typically observed at the polarised L|L interface (as described in detail in Figures 4 and 8). A symbol above the line represents an aqueous redox species A^w^ or D^w^, whereas a symbol below the line represents an organic redox species A^o^ or D^o^. Such schemes allow ease of visualisation of the relationships between $\Delta_{o}^{w}\varphi_{IET}^{app}$ , $\Delta_{o}^{w}\varphi_{IET}^{onset}$ , and $\eta_{EDL}$ , which are dependent on whether electrons flow from (a) the organic to aqueous phase or (b) the aqueous to organic phase, and whether $\Delta_{o}^{w}\varphi_{IET}^{onset}$ is (i) positive or (ii) negative of 0 V on the Galvani scale. $\Delta_{o}^{w}\varphi_{IET}^{onset}$ in turn is dependent on the nature of the ion distributions in the back‐to‐back EDLs as a function of the applied $\Delta_{o}^{w}\varphi$ . For example, if the redox species A^w^ or D^w^ is cationic then $\Delta_{o}^{w}\varphi_{IET}^{onset}$ will be in the potential range positive of the PZC [yellow shaded areas in (a)(i) and (b)(i)], while if A^w^ or D^w^ is anionic then $\Delta_{o}^{w}\varphi_{IET}^{onset}$ will be in the potential range negative of the PZC [yellow shaded areas in (a)(ii) and (b)(ii)]. The flow of electrons from the organic to the aqueous phase is spontaneous when $\Delta_{o}^{w}\varphi_{IET}^{app}<0\;\;V$ on the Galvani scale [blue shaded areas in (a)(i) and (a)(ii)]. The flow of electrons from the aqueous to the organic phase is spontaneous when $\Delta_{o}^{w}\varphi_{IET}^{app}>0\;\;V$ [blue shaded areas in (b)(i) and (b)(ii)]. $\Delta_{o}^{w}\varphi_{IET}^{app}$ can have a value outside the PPW.

**Figure 10:**
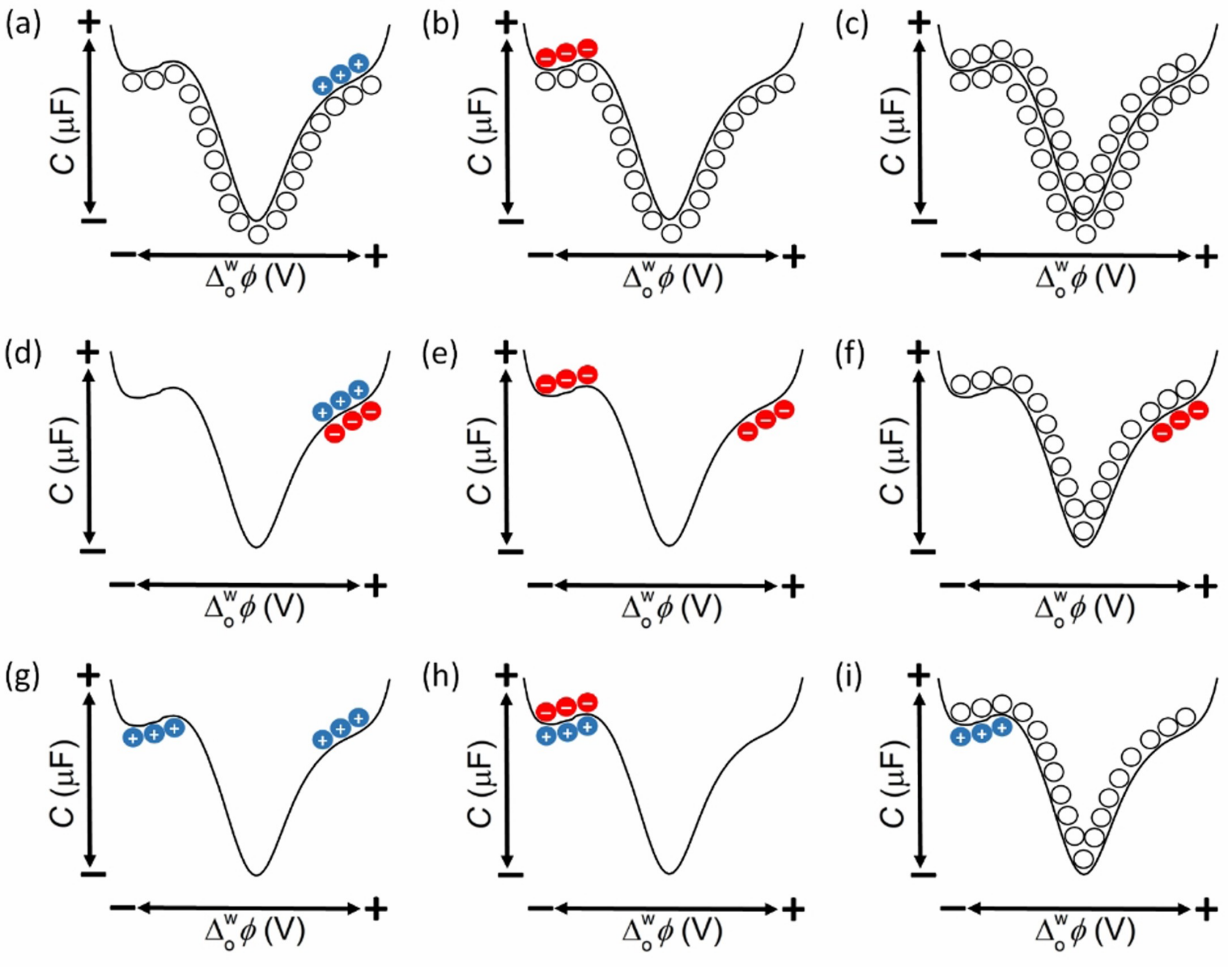
Schematic of the 9 possible distributions of the redox species in either side of the polarised L|L interface as a function of the applied $\Delta_{o}^{w}\varphi$ prior to the biphasic single‐step IET reaction. A list of aqueous and organic redox species that could correspond to most of these panels is provided in Table S4.

The reductant in the organic phase, D^o^, in Figure 9a and the acceptor in the organic phase, A^o^ in Figure 9b are chosen as neutral species. The oxidant in the aqueous phase, A^w^, in Figure 9a and the reductant in the aqueous phase, D^w^, in Figure 9b may be cationic [Figures 9a(i) and 9b(i)] or anionic species [Figures 9a(ii) and 9b(ii)]. However, this is just a snapshot of the possibilities, as each of the aqueous (D^w^ or A^w^) and organic (D^o^ and A^o^) redox species could be neutral, cationic, or anionic. In total, there are 9 possible ionic distributions of the redox species either side of the L|L interface as a function of the applied $\Delta_{o}^{w}\varphi$ prior to the biphasic single‐step IET reaction, as outlined in more detail *vide infra* when discussing Figure 10. For cationic or anionic redox species, interfacial accumulation will take place to compensate the build‐up of oppositely charged redox species and/or oppositely charged electrolyte ions within specific potential ranges. As a result, the interfacial concentration of the ionically charged redox species within these specific potential ranges may be orders of magnitude larger than their bulk phase concentrations depending on factors such as the charge of the ions (cationic, dicationic, etc.) and the interfacial surface area. The latter varies as a function of the applied $\Delta_{o}^{w}\varphi$ , notably increasing at the positive and negative extremes of the PPW. For neutral redox species, their distribution is unaffected by the applied $\Delta_{o}^{w}\varphi$ and their interfacial concentrations are homogeneous over the full PPW but at a level closer to the bulk phase concentrations than is the case for any ionically charged redox species.

In the schemes described in Figure 9, the PZC is arbitrarily set at a potential positive of 0 V on the Galvani scale and only thermodynamically spontaneous biphasic IET reactions are observable at applied $\Delta_{o}^{w}\varphi$ values greater or less than 0 V depending on the direction of the flow of electrons across the L|L interface (blue shaded areas in Figure 9). When the thermodynamically spontaneous flow of electrons is from a reductant in the organic phase (D^o+^/D^o^) to an oxidant in the aqueous phase (A^w−^/A^w^) redox couple, then $\eta_{EDL}$ >0 V if the potential range where A^w^ accumulates (yellow shaded areas in Figure 9a), and thus $\Delta_{o}^{w}\varphi_{IET}^{onset}$ , are at applied $\Delta_{o}^{w}\varphi$ values >0 V. This occurs when A^w^ is a cationic species as shown in Figure 9a(i), as is the case for the biphasic 2e^−^ ORR with DcMFc. On the other hand, if A^w^ is an anionic species and accumulates in a potential range <0 V, then $\eta_{EDL}\approx 0\;\;V$ or >0 V as shown in Figure 9a(ii). The former situation arises if $\Delta_{o}^{w}\varphi_{IET}^{onset}\approx \Delta_{o}^{w}\varphi_{IET}^{app}$ , as may occur within the potential range where the yellow and blue shaded bands overlap. When the thermodynamically spontaneous flow of electrons is from a reductant in the aqueous phase (D^w+^/D^w^) to oxidant in the organic phase (A^o−^/A^o^) redox couple, if the potential range where D^w^ accumulates is at applied $\Delta_{o}^{w}\varphi$ values >0 V, then $\eta_{EDL}\approx 0\;\;V$ if $\Delta_{o}^{w}\varphi_{IET}^{onset}\approx \Delta_{o}^{w}\varphi_{IET}^{app}$ , or $\eta_{EDL}$ >0 V as shown in Figure 9b(i). Finally, if the potential range where D^w^ accumulates is at applied $\Delta_{o}^{w}\varphi$ values <0, then $\eta_{EDL}$ >0 V as shown in Figure 9b(ii).

**Figure 11:**
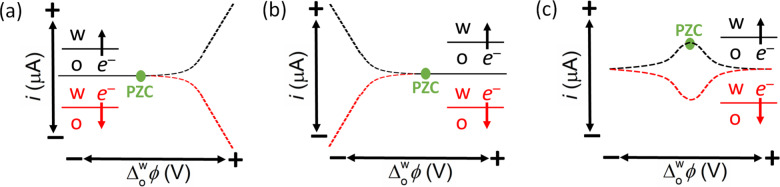
Schematic of the different current responses possible for biphasic single‐step IET reactions depending on the nature of the ionic distributions of the redox species either side of the polarised L|L interface as a function of the applied $\Delta_{o}^{w}\varphi$ prior to the biphasic single‐step IET reaction (as described in Figure 10). By convention, a current is positive if electrons flow from the organic to the aqueous phase (black lines) and negative if electrons flow from the aqueous to the organic phase (red lines). These idealised current profiles exclude any possible interferences from ion transfer of the products of the biphasic single‐step IET reaction.

A series of predictions can be made by careful consideration of the schemes in Figures 9 and 10. While this is a general discussion, a list of aqueous and organic soluble redox couples that could fulfil the roles of the redox species in the majority of the panels in Figure 10 is provided in Table S4. For the biphasic systems described in Figures 10a, d and f, $\Delta_{o}^{w}\varphi_{IET}^{onset}$ will always be positive of the PZC, with either a positive or negative current observed if electrons flow from the organic to aqueous phase or *vice versa*, respectively, as shown in Figure 11a. An interesting consequence is that the electron transfer from a reductant in the organic phase to an oxidant in the aqueous phase may be experimentally observed only at a positive applied $\Delta_{o}^{w}\varphi$ within the PPW, even if $\Delta_{o}^{w}\varphi_{IET}^{0}$ and $\Delta_{o}^{w}\varphi_{IET}^{app}$ are outside the negative edge of the PPW [as shown in Figure 9a(i)] and the reaction should take place when $\Delta_{o}^{w}\varphi >\Delta_{o}^{w}\varphi_{IET}^{app}$ , a range that includes negative applied $\Delta_{o}^{w}\varphi$ values. This is because the reaction is kinetically inhibited until the applied $\Delta_{o}^{w}\varphi$ is positive enough to overcome $\eta_{EDL}$ . This exact scenario was observed in our data, for example for the biphasic 2e^−^ ORR with DcMFc at pH 0.55, $\Delta_{o}^{w}\varphi_{IET}^{0}$ was determined as −0.555 V, far outside the negative limit of the PPW of ca. −0.400 V for a polarised aqueous|TFT interface, but $\Delta_{o}^{w}\varphi_{IET}^{onset}$ was experimentally observed at +0.342 V to overcome the large $\eta_{EDL}$ of 0.897 V.

For the biphasic systems described in Figures 10b, h and i, all of the trends are effectively the opposite of those described for Figures 10a, d and f. $\Delta_{o}^{w}\varphi_{IET}^{onset}$ will always be negative of the PZC, with either a positive or negative current observed, as shown in Figure 11(b). Additionally, the electron transfer from a reductant in the aqueous phase to an oxidant in the organic phase may be experimentally observed only at a negative applied $\Delta_{o}^{w}\varphi$ within the PPW, even if $\Delta_{o}^{w}\varphi_{IET}^{app}$ is outside the positive edge of the PPW [as shown in Figure 9b(ii)] and the reaction should take place when $\Delta_{o}^{w}\varphi <\Delta_{o}^{w}\varphi_{IET}^{app}$ , a range that includes positive applied $\Delta_{o}^{w}\varphi$ values.

For the systems described in Figures 10e and g, the IET may be completely inhibited or proceed at a low rate due to an “opposite diffuse layer effect”, as previously described by Niu and co‐workers.^[20]^ Considering Figure 10e, the anionic organic redox species accumulate in the (organic side of) EDL at positive $\Delta_{o}^{w}\varphi$ , while the anionic aqueous redox species accumulate in the (aqueous side of the) EDL at negative $\Delta_{o}^{w}\varphi$ . Thus, at applied $\Delta_{o}^{w}\varphi$ values ca. >200 mV either side of the PZC, one of the ionic redox species will have a substantially reduced or even negligible interfacial concentration (as modelled in Figure 7). Consequently, the optimal conditions for the biphasic IET reaction to proceed may be within a range of ca. 200 mV on either side of the PZC, where the interfacial concentration of one redox species is rapidly increasing and the other rapidly decreasing, though not yet at negligible levels. In these cases, a positive or negative current maximum, depending on the direction of the flow of electrons across the L|L interface, may be observed approaching the PZC as shown in Figure 11c. As Niu and co‐workers suggested,^[20]^ such a current maximum may easily be misinterpreted as evidence of “an inverted Marcus region”. From an experimental point of view, having a neutral redox species on either side of the L|L interface eliminates the complications that arise due to the “opposite diffuse layer effect”. Thus, it is of little surprise that the vast majority of biphasic single‐step IET reactions studied to date do indeed involve at least one neutral species (see Table S4), typically in the organic phase such as ferrocene derivatives or porphyrins as reductants and quinone derivatives as oxidants.

For the system described in Figure 10c, the biphasic single‐step IET reaction only deviates from proceeding at $\Delta_{o}^{w}\varphi_{IET}^{0}$ by a kinetic limitation related to $\eta_{R}$ . This is because the distributions of neutral redox species on either side of the L|L interface are unaffected by the applied $\Delta_{o}^{w}\varphi$ . In turn, as no $\Delta_{o}^{w}\varphi$ mediated increase in the interfacial concentrations of the neutral redox species is possible, the positive or negative current recorded may be lower than for similar experiments (in terms of ${\left[E_{{Ox}_{1}/{Red}_{1}}^{0}\right]}_{SHE}^{TFT}$ , ${\left[E_{{Ox}_{2}/{Red}_{2}}^{0}\right]}_{SHE}^{aq}$ and the bulk concentrations of the redox species in each phase) carried out with ionic redox species either on both sides (Figures 10d and h) or one side (Figures 10a, b, f and i) of the L|L interface.

On occasion, biphasic single‐step IET may be thermodynamically spontaneous at the equilibrium open circuit potential (OCP), i. e., without external polarisation of the L|L interface either by using a 4‐electrode electrochemical cell or by distribution of a common ion or salt. If such a biphasic single‐step IET reaction is desired, simply knowing if the equilibrium OCP is more positive or negative than $\Delta_{o}^{w}\varphi_{IET}^{0}$ is a poor guide in many cases to predict whether the reaction will proceed spontaneously within the PPW. In short, $\Delta_{o}^{w}\varphi_{IET}^{0}$ may be a reasonable predictor for those biphasic systems described in Figure 10 carried out either with neutral species on both sides of the L|L interface, or for those scenarios when $\eta_{EDL}\approx 0$ V with ionic redox species on both sides or one side of the L|L interface. However, $\Delta_{o}^{w}\varphi_{IET}^{0}$ is a poor predictor for those systems with ionic redox species requiring major redistributions of ions within the back‐to‐back EDLs to allow the biphasic single‐step IET reaction to proceed, in other words when $\eta_{EDL}>0\;\;V$ . It should be noted that the equilibrium OCP will not be constant with time during the biphasic single‐step IET reaction. As the reaction proceeds, the OCP will shift either positively or negatively due to the consumption of reactants and generation of products at the L|L interface and eventually move outside the optimal potential range for the biphasic single‐step IET reaction to proceed.

### Can varying the applied $\Delta_{o}^{w}\varphi$ drive a thermodynamically uphill biphasic single‐step IET reaction?

A key question is that if $\Delta_{o}^{w}\varphi_{IET}^{0}$ >0 V for a biphasic single‐step IET reaction where electrons flow from an organic to an aqueous redox couple, can varying the applied $\Delta_{o}^{w}\varphi$ drive such a thermodynamically uphill reaction under certain circumstances? Effectively, the answer is yes, but the applied $\Delta_{o}^{w}\varphi$ has an indirect influence on the biphasic single‐step IET reaction and not a direct influence as is the case at a solid electrolyte|electrolyte interface. The key is that $\Delta_{o}^{w}\varphi_{IET}^{0}$ is calculated using $E^{0}$ values determined for redox couples in the bulk aqueous and organic phases. However, the $E^{app}$ value for certain aqueous or organic redox couples in the mixed solvent region at the L|L interface may vary substantially from the $E^{0}$ value. This is because, as the applied $\Delta_{o}^{w}\varphi$ is scanned either positive or negative of the PZC, the environment experienced by both redox couples in the mixed solvent region changes compared to the bulk phases in terms of the dielectric constant, ionic strength and possibly pH. Thus, the biphasic single‐step IET reaction may proceed spontaneously in the mixed solvent region if $\Delta_{o}^{w}\varphi_{IET}^{app}<0\;\;V$ even though $\Delta_{o}^{w}\varphi_{IET}^{0}$ >0 V, which indicates that it is non‐spontaneous (under standard conditions).

The dielectric constant experienced in the mixed solvent region can be assumed to be an average of the dielectric constants of the aqueous and organic bulk phases. Thus, the dielectric constant experienced by the organic redox couple will increase compared to that in the bulk organic phase, while the dielectric constant experienced by the aqueous redox couple will decrease compared to that in the bulk aqueous phase. Such changes in dielectric constant change $E^{app}$ for certain redox couples. For example, the redox potential of the Fc^+^/Fc couple shifts to less positive potentials as the solvent dielectric constant increases, e. g., from +0.525 V [vs. Ag/AgCl/KCl (sat.)] in 1,2‐dichloroethane to +0.195 V [vs. Ag/AgCl/KCl (sat.)] in an aqueous solution.^[54]^ Meanwhile, the redox potential of the [Fe^(III)^(CN)_6_]^3−^/[Fe^(II)^(CN)_6_]^4−^ couple shifts to less positive potentials as the solvent dielectric constant decreases.^[55]^ On the other hand, the DcMFc redox potential is hardly affected by the nature of the solvent; therefore, our thermodynamic analyses herein are not affected by the nature of the mixed solvent region.^[54]^

As shown by our models of the interfacial concentrations of the aqueous and organic electrolyte cations and anions as a function of the applied $\Delta_{o}^{w}\varphi$ (Figure 7), the ionic strength in the mixed solvent region increases significantly scanning to applied $\Delta_{o}^{w}\varphi$ both positive and negative of the PZC. In this regard, the [Fe^(III)^(CN)_6_]^3−^/[Fe^(II)^(CN)_6_]^4−^ redox potential shifts to more positive potentials as the ionic strength increases.^[56]^ Thus, $E^{app}$ for the [Fe^(III)^(CN)_6_]^3−^/[Fe^(II)^(CN)_6_]^4−^ couple in the mixed solvent region is difficult to predict as any decreases of the redox potential due to the decreased dielectric constant may be compensated by increases of the redox potential due to increased ionic strength.

The values of $\Delta_{o}^{w}\varphi_{IET}^{0}$ reported for biphasic single‐step IET reactions between various aqueous and organic soluble redox couples are summarised in Table S4. In line with the key conclusion of the work herein, that the applied $\Delta_{o}^{w}\varphi$ provides no direct driving force to realise a thermodynamically uphill biphasic single‐step IET reaction in the mixed solvent region, all biphasic systems where electrons flow from the organic to aqueous phase report a “spontaneous” value of $\Delta_{o}^{w}\varphi_{IET}^{0}$ ≤0 V. The situation is less clear when electrons flow from the aqueous to organic phase, with several reports involving quinone derivatives or an oxidised organic soluble porphyrin (ZnPor^+^) as electron acceptors reporting a “non‐spontaneous” value of $\Delta_{o}^{w}\varphi_{IET}^{0}$ ≤0 V. One possible explanation is that $E^{app}$ for one or both of the redox couples in the mixed solvent region in these examples may vary substantially from their bulk $E^{0}$ values.

## Conclusion

The work herein supports the hypothesis that the applied $\Delta_{o}^{w}\varphi$ provides no direct driving force to realise a thermodynamically uphill biphasic single‐step IET reaction. Currents due to the biphasic 2e^−^ ORR were only recorded by cyclic voltammetry with DcMFc under thermodynamically spontaneous conditions at pH≤ 3. A key insight is that $\Delta_{o}^{w}\varphi_{IET}^{onset}$ does not correlate with the thermodynamically predicted $\Delta_{o}^{w}\varphi_{IET}^{0}$ , even if an intrinsic overpotential ($\eta_{R}$ ) of the biphasic 2e^−^ ORR due to reorganisation energies or double‐layer effects is considered. Instead, the biphasic IET reaction between DcMFc, O_2_ and interfacial protons is kinetically inhibited until the applied $\Delta_{o}^{w}\varphi$ is sufficiently positive to modulate the ion distribution at the polarised L|L interface such that the interfacial concentration of protons increases markedly. In this regard, we show that $\Delta_{o}^{w}\varphi_{IET}^{onset}\;$ is closely correlated with the PZC at a polarised L|L interface. An interesting consequence is that a biphasic single‐step IET reaction may be experimentally observed within the PPW, even if thermodynamically predicted to take place at an applied $\Delta_{o}^{w}\varphi$ far outside the PPW. Using a Verwey‐Niessen model of the polarised L|L interface, we calculate the interfacial concentrations of ions that accumulate in the back‐to‐back EDLs upon polarisation of the L|L interface. At an applied $\Delta_{o}^{w}\varphi$ positive of the PZC, we show that the concentration of interfacial protons is several orders of magnitude higher than the bulk aqueous phase concentration to compensate the build‐up of a concomitant interfacial concentration of organic electrolyte TB^−^ anions.

The experimental data provided herein is specific for a biphasic single‐step IET reaction between a cationic aqueous electron acceptor (interfacial protons) and a neutral organometallic electron donor (DcMFc). However, for other biphasic single‐step IET reactions, each of the aqueous and organic redox species could be neutral, cationic or anionic, and act as either the electron donor or acceptor. A general discussion of the nine possible ionic distributions of the redox species either side of the L|L interface as a function of the applied $\Delta_{o}^{w}\varphi$ prior to the biphasic single‐step IET reaction highlights that most studies to date use a neutral redox species in the organic phase to eliminate complications that arise due to the “opposite diffuse layer effect”. Finally, we outline that under certain circumstances the applied $\Delta_{o}^{w}\varphi$ can indirectly drive a biphasic single‐step IET reaction predicted to be thermodynamically uphill as the apparent redox potential $E^{app}$ for some aqueous or organic redox couples in the mixed solvent region may vary substantially from their bulk $E^{0}$ values.

The key objective of this article is to highlight that for applications, such as electrosynthesis, electrocatalysis, bioelectrochemistry, photoelectrochemistry, etc., based on biphasic single‐step IET reactions at a polarised L|L interface, the experimental design must consider both the thermodynamic spontaneity of IET between the aqueous and organic redox species in the mixed solvent region and the applied $\Delta_{o}^{w}\varphi$ required to modulate the interfacial ion distributions such that the aqueous and organic redox species are present in substantial concentrations at the L|L interface simultaneously in order to react.

## Experimental Section

### Chemicals

All chemicals were used as received without further purification. All aqueous solutions were prepared with ultrapure water (Millipore Milli‐Q, specific resistivity 18.2 MΩ ⋅ cm). Bis(triphenylphosphoranylidene) ammonium chloride (BACl, 97 %) and lithium tetrakis(pentafluorophenyl)borate diethyletherate (LiTB) were obtained from Sigma‐Aldrich and Boulder Scientific Company, respectively. Bis(triphenylphosphoranylidene) ammonium tetrakis (pentafluorophenyl)borate (BATB) was prepared by metathesis of equimolar solutions of BACl and LiTB in a methanol‐water (2 : 1 v/v) mixture. The resulting precipitates were filtered, washed, recrystallised from acetone and finally washed 5 times with methanol‐water (2 : 1 v/v) mixture. The organometallic electron donors, decamethylferrocene (DcMFc, ≥97 %) and 1,1’‐dimethylferrocene (DiMFc, >99 %), were used as obtained from Sigma‐Aldrich. Pentamethylferrocene (PMFc) was prepared by a minor variation of the literature route,^[57]^ as described below. Lithium chloride (LiCl, ≥95 %), lithium hydroxide (LiOH, 98 %), tetraethylammonium chloride (TEACl, ≥98 %), potassium dihydrogen phosphate (KH_2_PO_4_, ≥99 %), sodium hydrogen phosphate (Na_2_HPO_4_, ≥99 %) and sulfuric acid (H_2_SO_4_, 95–98 %) were obtained from Sigma‐Aldrich. The organic solvent α,α,α‐trifluorotoluene (TFT, 99 %) was obtained from both Alfa Aesar and Sigma‐Aldrich.

### Synthesis of pentamethylferrocene (PMFc)

All operations were conducted using Schlenk techniques under inert nitrogen atmosphere. Anhydrous FeCl_2_ was initially prepared by treating a suspension of commercial FeCl_2_.4H_2_O in hexane with 20 equivalents of trimethylsilyl chloride (SiClMe_3_). The suspension was heated to reflux for three hours (CARE – liberation of HCl), after which time the reaction was Schlenk filtered and the solid obtained washed with dry hexane. The anhydrous FeCl_2_ so obtained could be stored under dry nitrogen at ambient temperature.

A Schlenk flask charged with anhydrous FeCl_2_ (1.54 g, 12.1 mmol) and a magnetic stirrer bar was shielded from the light before being treated with THF (30 mL). The suspension was vigorously stirred for 1 hr at room temperature to give a green solution of FeCl_2_.2THF.^[58]^ After this time, the FeCl_2_.2THF solution was rapidly transferred by cannula into a second Schlenk flask containing a slurry of LiCp* (1.72 g, 12.1 mmol)^[59]^ in THF (80 mL) and the reaction mixture allowed to stir for a further hour at room temperature. A solution of LiCp (0.872 g, 12.11 mmol)^[59]^ in THF (50 mL) was slowly added to the mixture in the main flask containing [{FeCp*}_2_(μ‐Cl)_2_] and the reaction allowed to stir overnight. The following morning, the reaction solvent was removed under vacuum and the residue extracted into diethyl ether (50 mL). The extracts were filtered, and the solvent removed to give the crude product, which was purified by crystallisation from methanol (0.63 g, 20 %). Spectroscopic data (Figure S11–S13) were fully consistent with the available literature data.^[58]^

### Electrochemical measurements

Electrochemical experiments were carried out at an interface between two immiscible electrolyte solutions (ITIES) formed between water and TFT using a four‐electrode configuration (the geometric area of the cell was 1.53 cm^2^). To supply the current flow, platinum counter electrodes were positioned in the organic and aqueous phases. The potential drop at the liquid|liquid (L|L) interface was measured by means of pseudo‐reference silver/silver salt (Ag/AgX) electrodes, which were connected to the aqueous and organic phases, respectively, through Luggin capillaries (where X is the anion with the highest concentration in the aqueous phase, typically SO_4_^2−^ or Cl^−^ herein, to ensure reference potential stability). The general configurations of the four‐electrode electrochemical cells studied are outlined in Scheme 2, where each vertical line represents a phase boundary, and the double vertical line represents the polarisable L|L interface. All electrochemical measurements were carried out with a WaveDriver 20 bipotentiostat from Pine Research Instrumentation, Inc. and controlled by AfterMath software version 1.4. The applied potential (*E*) is related to the Galvani potential scale by the relationship: $E={\Delta_{o}^{w}\varphi +\Delta E}_{ref.}$ , where $\Delta_{o}^{w}\varphi$ is the interfacial Galvani potential difference and ${\Delta E}_{ref.}$ is the offset potential at each pH.^[60]^ The offset was estimated by assuming the formal ion transfer potential of the reference ion‐probe TEA^+^ to be +0.149 V at a polarised aqueous|TFT interface.^[37]^ All electrochemical measurements were carried out under ambient, aerobic conditions.

## Conflict of interest

The authors declare no conflict of interest.

1

## Supporting information

As a service to our authors and readers, this journal provides supporting information supplied by the authors. Such materials are peer reviewed and may be re‐organized for online delivery, but are not copy‐edited or typeset. Technical support issues arising from supporting information (other than missing files) should be addressed to the authors.

Supporting Information

## Data Availability

The data that support the findings of this study are available from the corresponding author upon reasonable request.
