# Supplementary material for: Potential‐Modulated Ion Distributions in the Back‐to‐Back Electrical Double Layers at a Polarised Liquid|Liquid Interface Regulate the Kinetics of Interfacial Electron Transfer
Source: ChemElectroChem. 2022 Dec 27;10(3):e202201042. doi: 10.1002/celc.202201042 (PMC10108062; doi:10.1002/celc.202201042)
Supplement: Supplementary file 1 — Supporting Information [file CELC-10-0-s001.pdf]

ChemElectroChem

Supporting Information

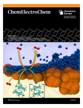

Potential-Modulated Ion Distributions in the Back-to-Back Electrical Double Layers at a Polarised Liquid|Liquid Interface Regulate the Kinetics of Interfacial Electron Transfer

Alonso Gamero-Quijano,* José A. Manzanares, Seyed M. B. H. Ghazvini, Paul J. Low, and Micheál D. Scanlon*

Table S1. Summary of the reduction half-reactions of (i) various aqueous, organic or interfacial O_2 or proton reduction reactions and (ii) various organic electron donor species, described in Figures 1a and b, and their associated standard redox potentials (expressed *versus* the standard hydrogen electrode (SHE)) as a function of pH.

Reduction Half-Reactions	Standard redox potentials (V)		pH	Ref.
$DcMFc^{+,TFT} + e^- \rightleftharpoons DcMFc^{TFT}$ [a]	$\left[E^0_{DcMFc^+/DcMFc}\right]^{TFT}_{SHE}$	0.107	n/a	[60]
$PMFc^{+,TFT} + e^- \rightleftharpoons PMFc^{TFT}$	$\left[E^0_{PMFc^+/PMFc}\right]^{TFT}_{SHE}$	0.415	n/a	This work (Figure S1)
$DiMFc^{+,TFT} + e^- \rightleftharpoons DiMFc^{TFT}$	$\left[E^0_{DiMFc^+/DiMFc}\right]^{TFT}_{SHE}$	0.617	n/a	This work (Figure S1)
$O_{2(g)} + 2H_3O^{+,aq} + 2e^- \rightleftharpoons H_2O_{2(l)} + 2H_2O_{(l)}$ (2e⁻ ORR) [b]	$\left[E^0_{O_2/H_2O_2}\right]^{aq}_{SHE}$	0.665	0.5	[61]
		0.577	2	
		0.429	4.5	
		0.281	7	
		0.104	10	
		–0.014	12	
$O_{2(g)} + 4H_3O^{+,aq} + 4e^- \rightleftharpoons 6H_2O_{(l)}$ (4e⁻ ORR) [c]	$\left[E^0_{O_2/H_2O}\right]^{aq}_{SHE}$	1.199	0.5	[61]
		1.111	2	
		0.964	4.5	
		0.815	7	
		0.638	10	
		0.520	12	
$O_2^{\bullet-,aq} + H_3O^{+,aq} \rightleftharpoons HO_2^{\bullet,aq} + H_2O_{(l)}$ (perhydroxyl radical) [d]	$\left[E^0_{O_2^{\bullet-}/HO_2^{\bullet}}\right]^{aq}_{SHE}$	0.094	0.5	[62]
		0.005	2	
		–0.143	4.5	
$O_{2(g)} + e^- \rightleftharpoons O_2^{\bullet-,aq}$ (superoxide radical anion)	$\left[E^0_{O_2/O_2^{\bullet-}}\right]^{aq}_{SHE}$	–0.160	7	
		–0.160	10	
		–0.160	12	
$2H_3O^{+,aq} + 2e^- \rightleftharpoons H_{2(g)} + 2H_2O_{(l)}$ [e]	$\left[E^0_{H_3O^+/\frac{1}{2}H_2}\right]^{aq}_{SHE}$	–0.030	0.5	[63]
		–0.118	2	
		–0.266	4.5	
		–0.414	7	
		–0.591	10	
		–0.709	12	
$2H^{+,TFT} + 2e^- \rightleftharpoons H_{2(g)}$	$\left[E^0_{H^+/\frac{1}{2}H_2}\right]^{TFT}_{SHE}$	0.717	n/a	[63]
$2[H^+ \ldots TB^-]^{int} + 2e^- \rightleftharpoons H_{2(g)} + 2TB^{-,int}$ [f]	$\left[E^0_{[H^+ \ldots TB^-]/\frac{1}{2}H_2}\right]^{int}_{SHE}$	0.344	0.5	This work
		0.299	2	
		0.225	4.5	
		0.151	7	
		0.063	10	
		0.004	12	

[a] Note that the redox potential of the $DcMFc^+/DcMFc$ redox couple is barely affected by the solvent compositions and can be considered constant in the mixed solvent region. Thus, DcMFc should be considered a superior redox couple for studying solvent effects on the thermodynamics of electron transfer

reactions at aqueous|TFT interfaces.[54] In contrast, the standard redox potentials of DiMFc and PMFc may be affected by the composition of the mixed solvent layer.

[b] $\left[E^0_{O_2/H_2O_2}\right]^{aq.}_{SHE} = 0.695 - 0.0591\mathrm{pH}$

[c] $\left[E^0_{O_2/H_2O}\right]^{aq.}_{SHE} = 1.229 - 0.0591\mathrm{pH}$

[d] The superoxide radical anion ($O_2^{\bullet-,aq}$) has a pK_a of 4.8 and at pH < 4.8 it is protonated forming perhydroxyl radicals ($HO_2^{\bullet,aq}$).[64] Thus, the standard redox potential of $HO_2^{\bullet,aq}$ is pH-dependent according to the relationship: $\left[E^0_{O_2^{\bullet-}/HO_2^{\bullet}}\right]^{aq}_{SHE} = -0.160 - 0.0591(\mathrm{pH} - \mathrm{p}K_a)$. Thus, at pH = 4.8 $\left[E^0_{O_2^{\bullet-}/HO_2^{\bullet}}\right]^{aq}_{SHE} = -0.160$ and at pH = 2 $\left[E^0_{O_2^{\bullet-}/HO_2^{\bullet}}\right]^{aq}_{SHE} = -0.160 - 0.0591 \times (-2.8) = 0.005$ V.

[e] $\left[E^0_{H_3O^+/\frac{1}{2}H_2}\right]^{aq}_{SHE} = 0 - 0.0591\mathrm{pH}$

[f] $\left[E^0_{[H^+\ldots TB^-]/\frac{1}{2}H_2}\right]^{int.}_{SHE} = \frac{1}{2}\left(\left[E^0_{H_3O^+/\frac{1}{2}H_2}\right]^{aq}_{SHE} + \left[E^0_{H^+/\frac{1}{2}H_2}\right]^{TFT}_{SHE}\right)$

Table S2. Summary of the interfacial electron transfer (IET) reactions for biphasic ORRs (2e$^-$ or 4e$^-$ pathways) with various organic electron donors, described in Figure 1c and Figure S2a, and their associated standard Galvani IET potentials $\left(\Delta_{o}^{w}\phi_{IET}^{0}\right)$ as a function of pH.

IET reaction	Standard Galvani IET potential (V)		pH
$2DcMFc^{TFT} + O_{2(g)} + 2H_3O^{+,aq} \rightleftharpoons 2DcMFc^{+,TFT} + H_2O_{2(l)} + 2H_2O_{(l)}$	$\Delta_{o}^{w}\phi_{IET}^{0}(O_2\|\|DcMFc\ (2e^-\ ORR)) = \left[E_{DcMFc^+/DcMFc}^{0}\right]_{SHE}^{TFT} - \left[E_{O_2/H_2O_2}^{0}\right]_{SHE}^{aq}$	–0.558	0.5
		–0.470	2
		–0.322	4.5
		–0.174	7
		0.003	10
		0.121	12
$4DcMFc^{TFT} + O_{2(g)} + 4H_3O^{+,aq} \rightleftharpoons 4DcMFc^{+,TFT} + 6H_2O_{(l)}$	$\Delta_{o}^{w}\phi_{IET}^{0}(O_2\|\|DcMFc\ (4e^-\ ORR)) = \left[E_{DcMFc^+/DcMFc}^{0}\right]_{SHE}^{TFT} - \left[E_{O_2/H_2O}^{0}\right]_{SHE}^{aq}$	–1.092	0.5
		–1.004	2
		–0.857	4.5
		–0.708	7
		–0.531	10
		–0.413	12
$2PMFc^{TFT} + O_{2(g)} + 2H_3O^{+,aq} \rightleftharpoons 2PMFc^{+,TFT} + H_2O_{2(l)} + 2H_2O_{(l)}$	$\Delta_{o}^{w}\phi_{IET}^{0}(O_2\|\|PMFc\ (2e^-\ ORR)) = \left[E_{PMFc^+/PMFc}^{0}\right]_{SHE}^{TFT} - \left[E_{O_2/H_2O_2}^{0}\right]_{SHE}^{aq}$	–0.250	0.5
		–0.162	2
		–0.014	4.5
		0.134	7
		0.311	10
		0.429	12
$4PMFc^{TFT} + O_{2(g)} + 4H_3O^{+,aq} \rightleftharpoons 4PMFc^{+,TFT} + 6H_2O_{(l)}$	$\Delta_{o}^{w}\phi_{IET}^{0}(O_2\|\|PMFc\ (4e^-\ ORR)) = \left[E_{PMFc^+/PMFc}^{0}\right]_{SHE}^{TFT} - \left[E_{O_2/H_2O}^{0}\right]_{SHE}^{aq}$	–0.784	0.5
		–0.696	2
		–0.549	4.5
		–0.400	7
		–0.223	10
		–0.105	12
$2DiMFc^{TFT} + O_{2(g)} + 2H_3O^{+,aq} \rightleftharpoons 2DiMFc^{+,TFT} + H_2O_{2(l)} + 2H_2O_{(l)}$	$\Delta_{o}^{w}\phi_{IET}^{0}(O_2\|\|DiMFc\ (2e^-\ ORR)) = \left[E_{DiMFc^+/DiMFc}^{0}\right]_{SHE}^{TFT} - \left[E_{O_2/H_2O_2}^{0}\right]_{SHE}^{aq}$	–0.048	0.5
		0.040	2
		0.188	4.5
		0.336	7
		0.513	10
		0.631	12
$4DiMFc^{TFT} + O_{2(g)} + 4H_3O^{+,aq} \rightleftharpoons 4DiMFc^{+,TFT} + 6H_2O_{(l)}$	$\Delta_{o}^{w}\phi_{IET}^{0}(O_2\|\|DiMFc\ (4e^-\ ORR)) = \left[E_{DiMFc^+/DiMFc}^{0}\right]_{SHE}^{TFT} - \left[E_{O_2/H_2O}^{0}\right]_{SHE}^{aq}$	–0.582	0.5
		–0.494	2
		–0.347	4.5
		–0.198	7
		–0.021	10
		0.097	12

Table S3. Summary of the IET reactions for biphasic reduction of aqueous (H_3O^+) or interfacial [H^+…TB^-] protons with various organic electron donors, described in Figure 1d and Figure S2b, and their associated standard Galvani IET potentials $\left(\Delta_o^w \phi_{IET}^0\right)$ as a function of pH.

IET reaction	Standard Galvani IET potential (V)		pH
$2DcMFc^{TFT} + 2H_3O^{+,aq} \rightleftharpoons 2DcMFc^{+,TFT} + H_{2(g)} + 2H_2O_{(l)}$	$\Delta_o^w \phi_{IET}^0(H_3O^+\vert\vert DcMFc) = \left[E^0_{DcMFc^+/DcMFc}\right]^{TFT}_{SHE} - \left[E^0_{H_3O^+/\frac{1}{2}H_2}\right]^{aq}_{SHE}$	0.137	0.5
		0.225	2
		0.373	4.5
		0.521	7
		0.698	10
		0.816	12
$2DcMFc^{TFT} + 2[H^+\ldots TB^-]^{int} \rightleftharpoons 2DcMFc^{+,TFT} + H_{2(g)} + 2TB^{-,int}$	$\Delta_o^w \phi_{IET}^0([H^+ \ldots TB^-]\vert\vert DcMFc) = \left[E^0_{DcMFc^+/DcMFc}\right]^{TFT}_{SHE} - \left[E^0_{[H^+\ldots TB^-]/\frac{1}{2}H_2}\right]^{int}_{SHE}$	–0.237	0.5
		–0.192	2
		–0.118	4.5
		–0.044	7
		0.044	10
		0.103	12
$2PMFc^{TFT} + 2H_3O^{+,aq} \rightleftharpoons 2PMFc^{+,TFT} + H_{2(g)} + 2H_2O_{(l)}$	$\Delta_o^w \phi_{IET}^0(H_3O^+\vert\vert PMFc) = \left[E^0_{PMFc^+/PMFc}\right]^{TFT}_{SHE} - \left[E^0_{H_3O^+/\frac{1}{2}H_2}\right]^{aq}_{SHE}$	0.445	0.5
		0.533	2
		0.671	4.5
		0.829	7
		1.006	10
		1.124	12
$2PMFc^{TFT} + 2[H^+\ldots TB^-]^{int} \rightleftharpoons 2PMFc^{+,TFT} + H_{2(g)} + 2TB^{-,int}$	$\Delta_o^w \phi_{IET}^0([H^+ \ldots TB^-]\vert\vert PMFc) = \left[E^0_{PMFc^+/PMFc}\right]^{TFT}_{SHE} - \left[E^0_{[H^+\ldots TB^-]/\frac{1}{2}H_2}\right]^{int}_{SHE}$	0.071	0.5
		0.116	2
		0.190	4.5
		0.264	7
		0.352	10
		0.411	12
$2DiMFc^{TFT} + 2H_3O^{+,aq} \rightleftharpoons 2DiMFc^{+,TFT} + H_{2(g)} + 2H_2O_{(l)}$	$\Delta_o^w \phi_{IET}^0(H_3O^+\vert\vert DiMFc) = \left[E^0_{DiMFc^+/DiMFc}\right]^{TFT}_{SHE} - \left[E^0_{H_3O^+/\frac{1}{2}H_2}\right]^{aq}_{SHE}$	0.647	0.5
		0.735	2
		0.883	4.5
		1.031	7
		1.208	10
		1.326	12
$2DiMFc^{TFT} + 2[H^+\ldots TB^-]^{int} \rightleftharpoons 2DiMFc^{+,TFT} + H_{2(g)} + 2TB^{-,int}$	$\Delta_o^w \phi_{IET}^0([H^+ \ldots TB^-]\vert\vert DiMFc) = \left[E^0_{DiMFc^+/DiMFc}\right]^{TFT}_{SHE} - \left[E^0_{[H^+\ldots TB^-]/\frac{1}{2}H_2}\right]^{int}_{SHE}$	0.273	0.5
		0.318	2
		0.392	4.5
		0.466	7
		0.554	10
		0.613	12

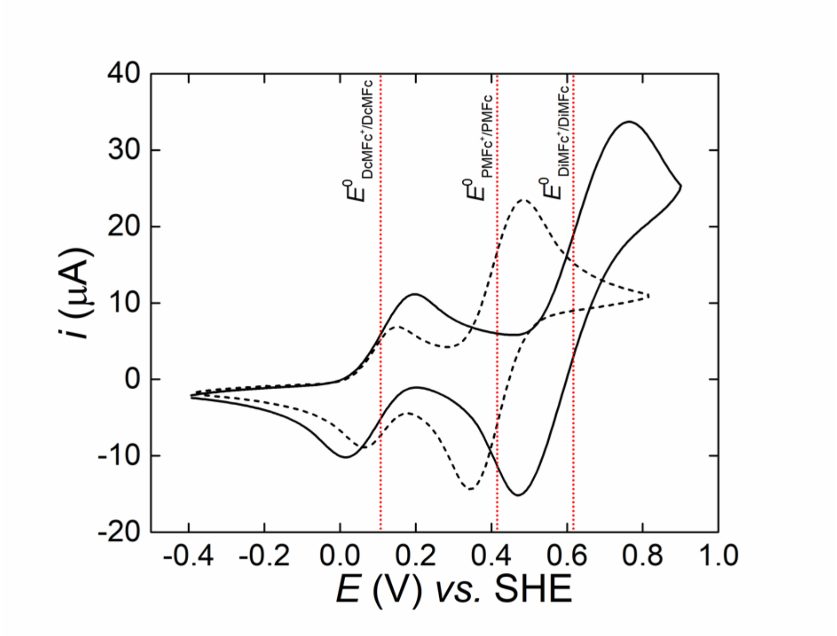

Figure S1. Cyclic voltammograms (CVs) of the reversible $DcMFc^+$/DcMFc, $PMFc^+$/PMFc and $DiMFc^+$/DiMFc redox couples in TFT. All CVs were calibrated to the standard hydrogen electrode (SHE) scale by using the $DcMFc^+$/DcMFc redox couple as an internal calibrant. The standard redox potential of the latter in TFT is 0.107 V *vs.* SHE (see Table S1). The solid black CV is of 110.3 μM DcMFc and 169.2 μM PMFc, and the dashed black CV is of 110.3 μM DcMFc and 234.0 μM DiMFc. The organic supporting electrolyte was 5 mM BATB. All CVs were obtained under a nitrogen atmosphere in a 3-electrode electrochemical cell with TFT solutions degassed for 15 min and at a scan rate of 20 mV s^{-1}. ITO and platinum were used as the working and counter electrodes respectively, while a Ag wire acted as the *pseudo*-reference electrode. The standard redox potentials of the $PMFc^+$/PMFc and $DiMFc^+$/DiMFc redox couples in TFT were determined as 0.415 and 0.617 V *vs.* SHE, respectively.

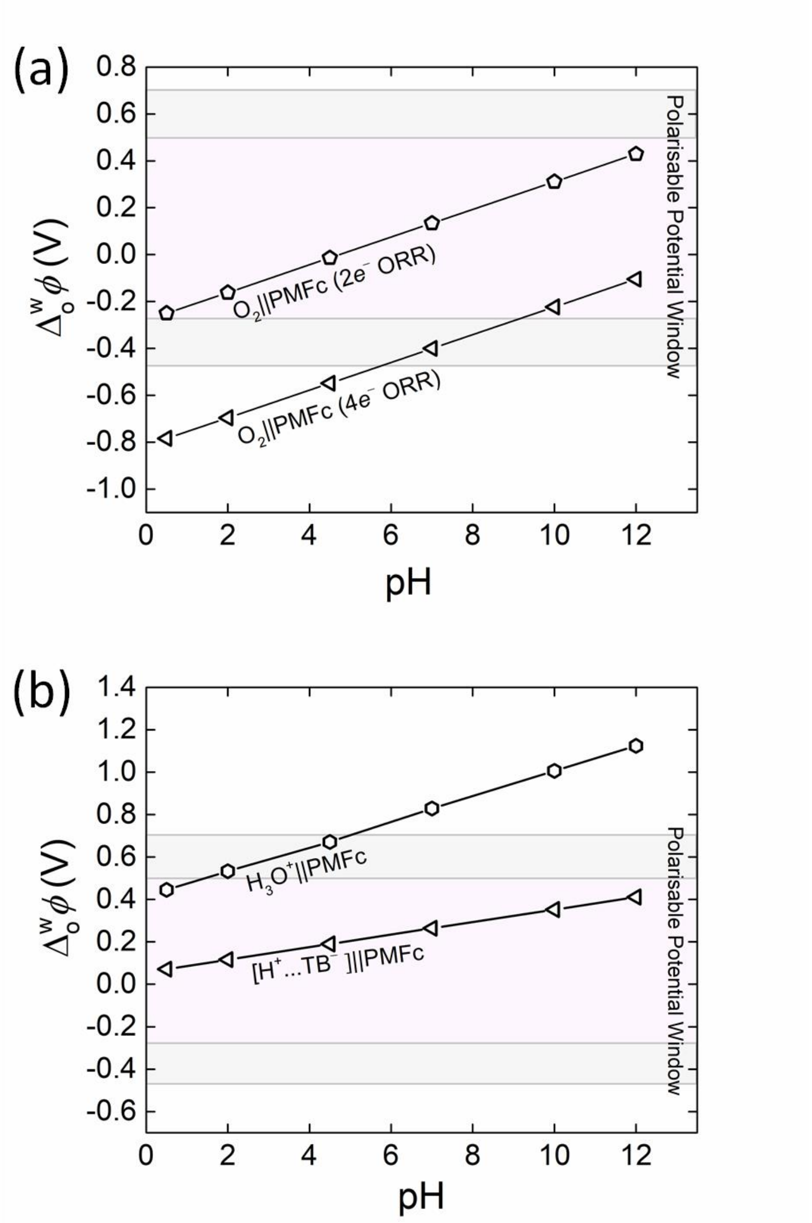

Figure S2. (a) Plot of $\Delta_o^w \phi_{IET}^0$ as a function of pH for the biphasic ORR ($2e^-$ or $4e^-$ pathways) with PMFc as the organic electron donor. (b) Plot of $\Delta_o^w \phi_{IET}^0$ as a function of pH for the reduction of aqueous (H_3O^+) and interfacial [H^+…TB^-] protons with PMFc as the organic electron donor. The sources of electrochemical data and equations used to construct plots (a) and (b) are described in Tables S2 and S3, respectively.

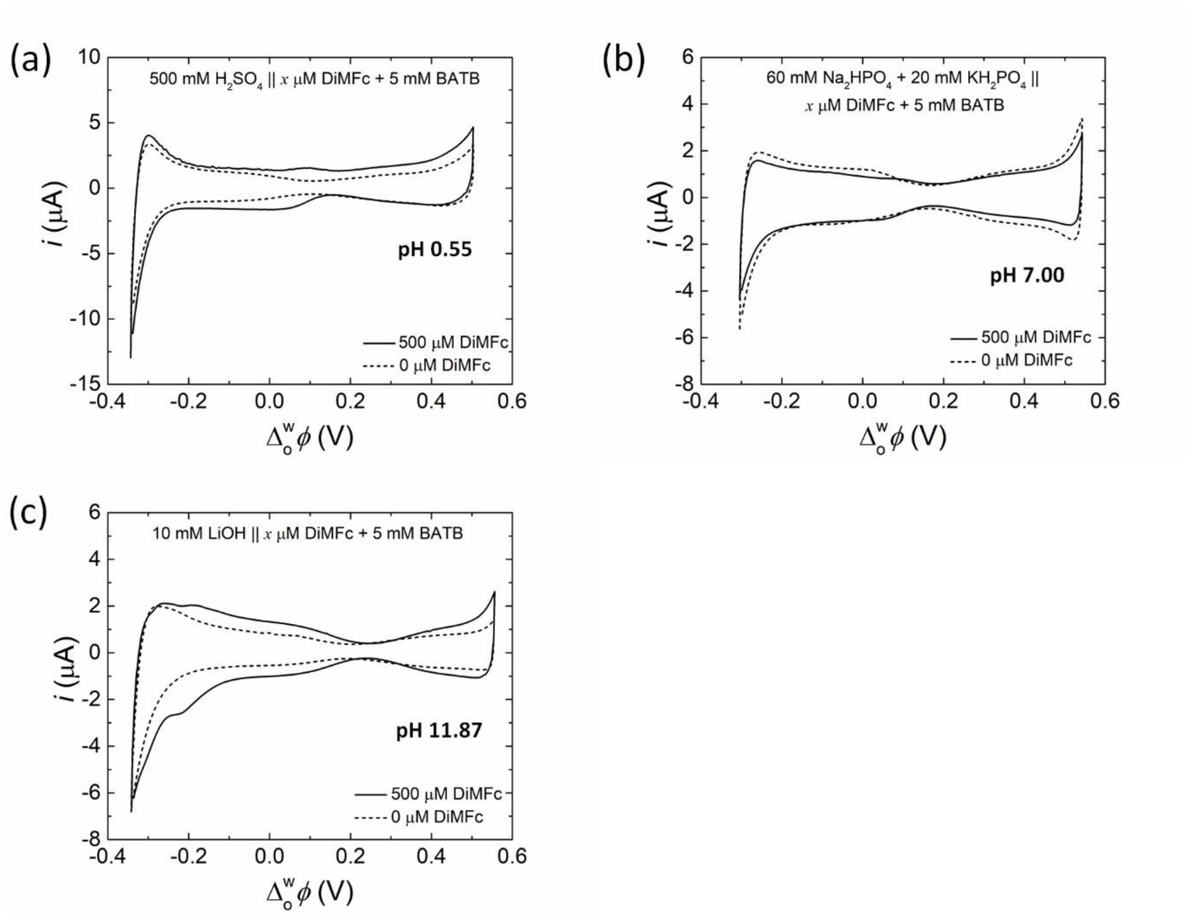

Figure S3. CVs obtained in the presence (solid) and absence (dashed) of 500 μM DiMFc at pH 0.55, 7.00 and 11.87, respectively. All CVs were obtained at a scan rate of 20 $mV\cdot s^{-1}$ using Electrochemical Cells 1 (for pH 0.55), 2 (for pH 7.00) and 3 (for pH 11.87), respectively, under aerobic, ambient conditions (see Scheme 2). The compositions of the aqueous and organic phases for each electrochemical cell are further noted in each panel.

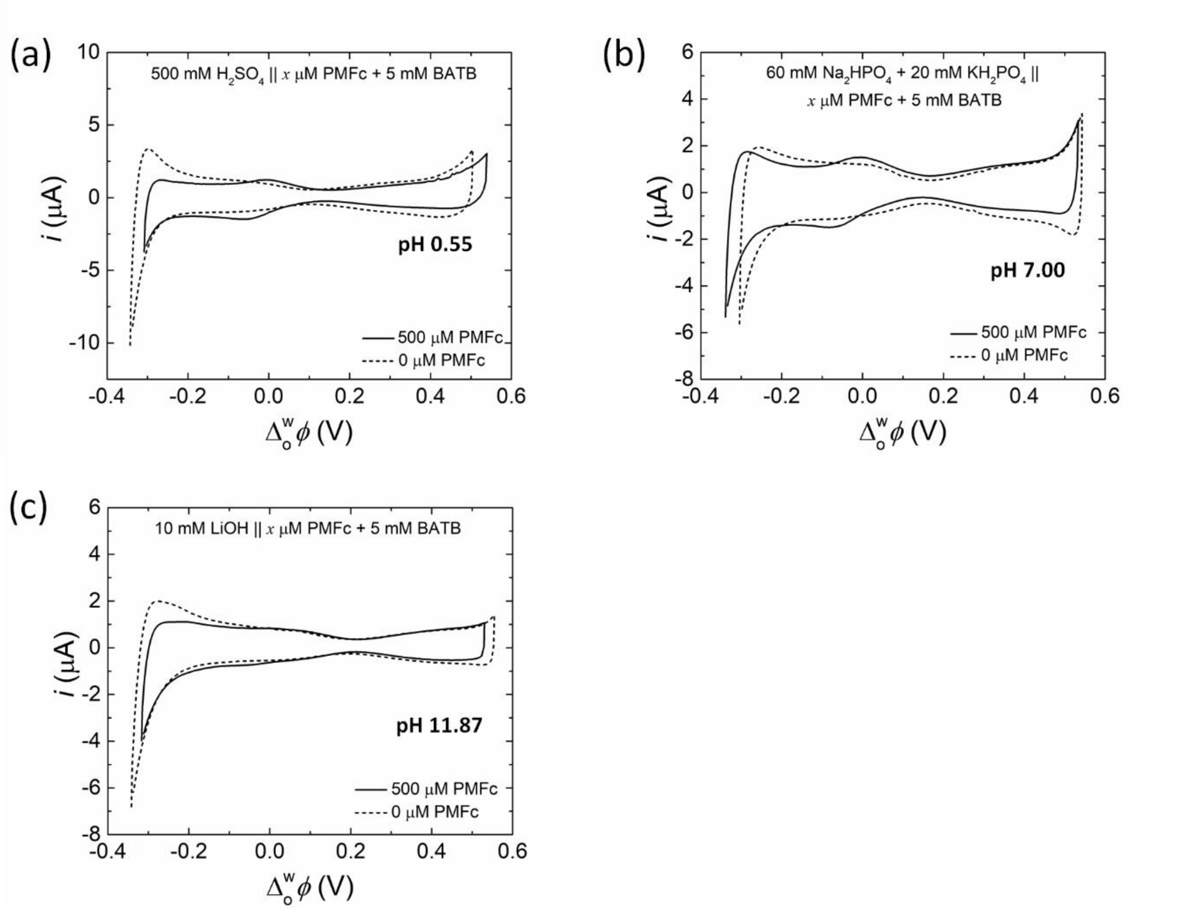

Figure S4. CVs obtained in the presence (solid) and absence (dashed) of 500 μM PMFc at pH 0.55, 7.00 and 11.87, respectively. All CVs were obtained at a scan rate of 20 mV·s^{-1} using Electrochemical Cells 1 (for pH 0.55), 2 (for pH 7.00) and 3 (for pH 11.87), respectively, under aerobic, ambient conditions (see Scheme 2). The compositions of the aqueous and organic phases for each electrochemical cell are further noted in each panel.

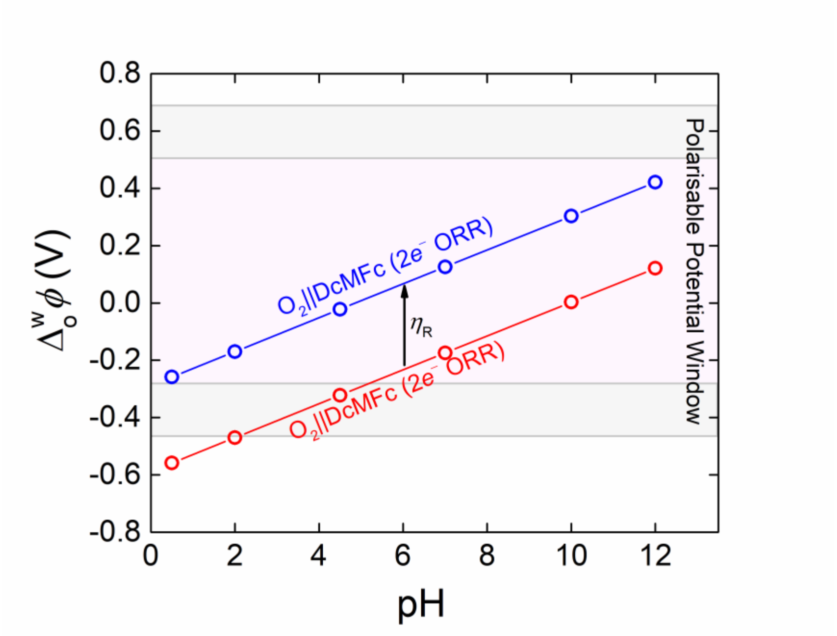

Figure S5. The apparent Galvani IET potential $\Delta_o^w\phi_{IET}^{app}$ (blue circles) for the biphasic 2e⁻ ORR with DcMFc shifts positively by η_R on the Galvani scale compared with $\Delta_o^w\phi_{IET}^0$ (red circles) as shown in a plot of $\Delta_o^w\phi$ *versus* pH.

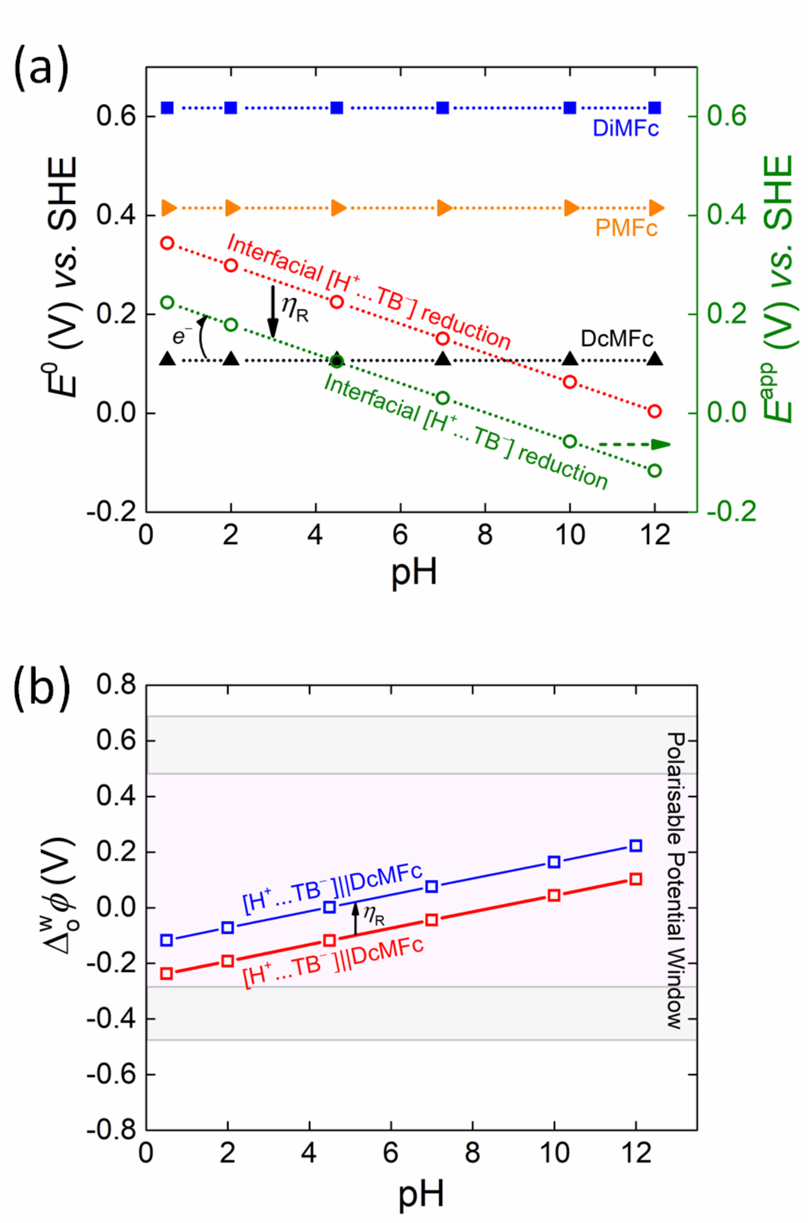

Figure S6. (a) Taking the intrinsic overpotential (η_R) of biphasic proton reduction at a polarised L|L interface into account, E^{app} (green circles) required to reduce interfacial [H^+…TB^-] protons shifts negatively compared with E^0 (red circles). (b) In turn, $\Delta_{\mathrm{o}}^{\mathrm{w}}\phi_{\mathrm{IET}}^{\mathrm{app}}$ (blue squares) for the biphasic reduction of interfacial [H^+…TB^-] protons with DcMFc shifts positively by η_{R} on the Galvani scale compared with $\Delta_{\mathrm{o}}^{\mathrm{w}}\phi_{\mathrm{IET}}^{0}$ (red squares) as shown in a plot of $\Delta_{\mathrm{o}}^{\mathrm{w}}\phi$ *versus* pH.

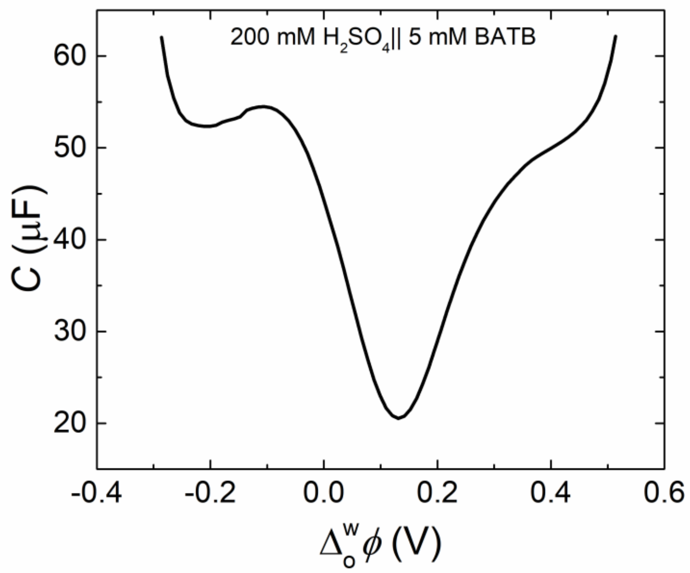

Figure S7. Differential capacitance measurement with an aqueous phase containing 200 mM H_2SO_4 and the TFT phase containing 5 mM BATB. The measurement was taken using a voltage excitation frequency of 5 Hz under aerobic, ambient conditions.

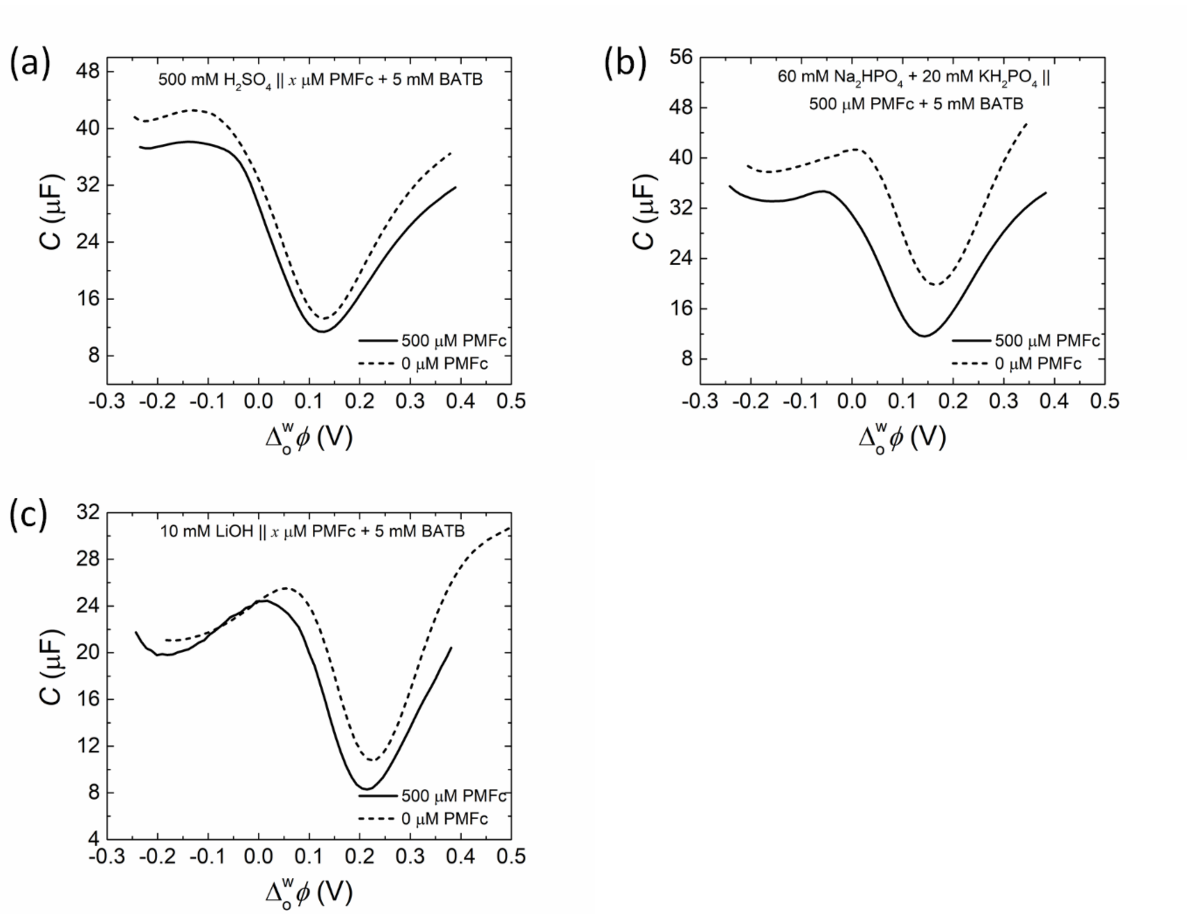

Figure S8. Differential capacitance measurements demonstrate that the PZC does not shift meaningfully in the presence of the neutral electron donor, in this case 500 μM PMFc, across the pH range (a) pH 0.55, (b) pH 7.00 and (c) pH 11.87. The magnitude of the interfacial capacitance does decrease in the presence of PMFc at each pH. Differential capacitance measurements were taken using a voltage excitation frequency of 5 Hz at pH 0.55 using Electrochemical Cell 1, at pH 7.00 using Electrochemical Cell 2 and at pH 11.87 using Electrochemical Cell 3 under aerobic, ambient conditions (see Scheme 2).

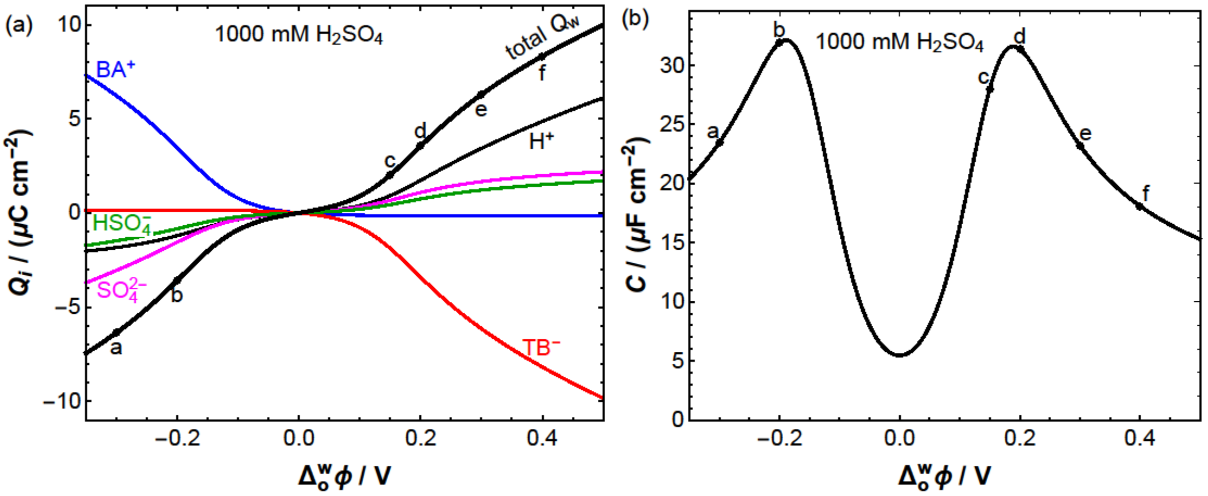

Figure S9. (a) Ionic contributions Q_i to the separated charge density across the L|L interface in Electrochemical Cell 1 (see Scheme 2a), 5mM BATB (TFT) || 1000 mM H_2SO_4 (aq), as a function of the applied interfacial Galvani potential difference ($\Delta_o^w\phi$). Note that $Q^w = Q_{H^+} + Q_{HSO_4^-} + Q_{SO_4^{2-}} = -Q_{BA^+} - Q_{TB^-} = -Q^o$. Labels a-f mark the potentials corresponding to the ionic concentration profiles shown in Figure S10. (b) The slope of the total Q^w curve is the differential capacitance.

The description of the dissociation of sulfuric acid into protons (subscript H), bisulfate ions (subscript 1) and sulfate ions (subscript 2) has to take into account the strong interactions between the ions. The dissociation equilibrium of bisulfate ions can be described by

$$K_d(c_0) = \frac{c_H c_2}{c_1} \tag{S1}$$

where the dissociation “constant” $K_d(c_0)$ is a function of the sulfuric acid concentration c_0. Since $c_0 = c_1 + c_2$ and $2c_0 = c_H + c_1$, we can introduce the fractions $x_1 = c_1/c_0$, $x_2 = c_2/c_0 = 1 - x_1$, $x_H = c_H/c_0 = 2 - x_1$, and rewrite Eq. (S1) as

$$k_d(c_0) \equiv \frac{K_d(c_0)}{c_0} = \frac{x_H x_2}{x_1} = \frac{(2-x_1)(1-x_1)}{x_1} \tag{S2}$$

and solve for the fraction of sulfuric acid molecules dissociated to bisulfate ions

$$x_1(c_0) = \frac{1}{2}\left(3 + k_d(c_0) - \sqrt{1 + 6k_d(c_0) + k_d(c_0)^2}\right). \tag{S3}$$

By fitting the experimental data in ref. [65] we find

$$k_d(c_0) = 0.1061\ (c_0/\mathrm{M})^{-1/2} + 0.3252 + 0.09296(c_0/\mathrm{M})\,. \tag{S4}$$

Thus, in the bulk, electroneutral aqueous solution with a sulfuric acid concentration c_0, the ionic concentrations are obtained as $c_H = [2 - x_1(c_0)]c_0$, $c_1 = x_1(c_0)c_0$ and $c_2 = [1 - x_1(c_0)]c_0$, where $x_1(c_0)$ is given by Eqs. (S3) and (S4).

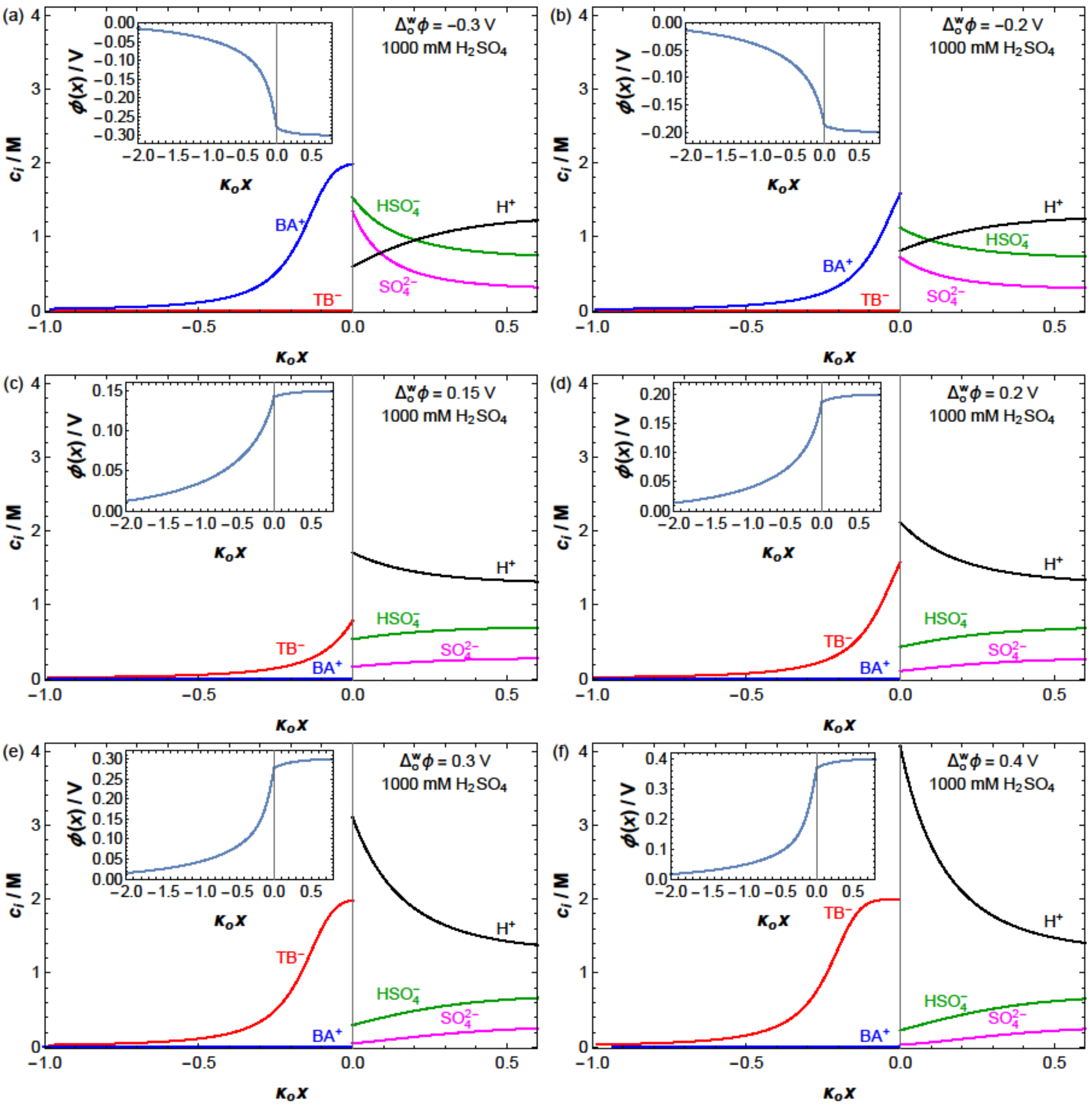

Figure S10. Ionic concentration profiles in Electrochemical Cell 1 (Scheme 2a), 5mM BATB (TFT) || 1000 mM H_2SO_4 (aq), for applied $\Delta_o^w\phi$ values of: (a) –0.3 V, (b) –0.2 V, (c) +0.15 V, (d) +0.2 V, (e) +0.3 V, and (f) +0.4 V. The maximum concentration of the organic ions due to their size has been given the roughly estimated value of 2 M. The L|L interface is located at x = 0. Position x has been scaled with the reciprocal Debye length κ_o in phase o ($1/\kappa_o = 1.489$ nm). The insets show the corresponding electrical potential profiles.

Table S4. A list of aqueous and organic soluble redox species that could fulfil the roles of the redox species in the majority of the panels shown in Figure 10. This table is not an exhaustive list, for example excluding photo-induced biphasic IET reactions, and simply contains representative examples.

**Charge of the aqueous and organic redox species prior to the biphasic IET reaction**		**Representative examples of biphasic IET reactions**		**Direction of biphasic IET**	**Ref.**	$\Delta_o^w \phi^0_{IET}$ **(V)**
**Aqueous redox species**	**Organic redox species**	**Aqueous redox species [a], [b]**	**Organic redox species [c],[d]**			
Cationic	Neutral	Protons (and O_2)	DcMFc	(o)→(w)	This work	–0.55 (pH 2; $2e^-$ ORR)
		Ce^{4+}	EDOT	(o)→(w)	[1]	0.00
		Ag^+	BuFc	(o)→(w)	[66]	–0.24
		Cu^{2+}	DcMFc	(o)→(w)	[67]	–0.30
Anionic	Neutral	$[Fe^{(III)}(CN)_6]^{3-}$	DcMFc	(o)→(w)	[68]	–0.30
		$[Ir^{(IV)}Cl_6]^{2-}$	DcMFc	(o)→(w)	[68]	–0.93
		$[Ru^{(III)}(CN)_6]^{3-}$	DcMFc	(o)→(w)	[68]	–0.79
		$[Ru^{(III)}(CN)_6]^{3-}$	ZnPor	(o)→(w)	[21]	–1.31
		$[Fe^{(II)}(CN)_6]^{4-}$	TCNQ	(w)→(o)	[69–71]	–0.15
		$[Fe^{(II)}(CN)_6]^{4-}$	TCBQ	(w)→(o)	[71]	–0.21
		$[Fe^{(II)}(CN)_6]^{4-}$	TFBQ	(w)→(o)	[71]	–0.26
		$[Fe^{(III)}(CN)_6]^{3-}$	TTF	(o)→(w)	[71,72]	+0.06
		$[Pt^{(II)}Cl_4]^{2-}$	DcMFc	(o)→(w)	[39,73]	–0.69
		$[Pd^{(II)}Cl_4]^{2-}$	DcMFc	(o)→(w)	[73–75]	–0.57
		$[Au^{(III)}Cl_4]^-$	TPTA	(o)→(w)	[76]	Not given
Neutral	Neutral	$Co^{(I)}L\text{-}H$	RBr_2	(w)→(o)	[77]	Not given
		NADH	TCBQ	(w)→(o)	[78]	+0.48
		NADH	MBQ	(w)→(o)	[78]	Not given
		H_2O_2	TCHQ	(w)→(o)	[79]	–0.85
Cationic	Anionic	No examples found				
Anionic	Anionic	$[Fe^{(III)}(CN)_6]^{3-}$	$TCNQ^-$	(o)→(w)	[80]	–0.15
		$[Fe^{(III)}(CN)_6]^{3-}$	C_{60}^-	(o)→(w)	[81]	–0.72
Neutral	Anionic	No examples found				
Cationic	Cationic	$[Ru^{(II)}(NH_3)_6]^{2+}$	$ZnPor^+$	(w)→(o)	[82]	–0.52
Anionic	Cationic	$[Fe^{(II)}(CN)_6]^{4-}$	Fc^+ or $DiMFc^+$	(w)→(o)	[83]	+0.17 or +0.05
		$[Ru^{(II)}(CN)_6]^{4-}$	$ZnPor^+$	(w)→(o)	[84]	–1.31
Neutral	Cationic	No examples found				

[a] Aqueous soluble oxidants

Neutral: No example found

Cationic: Protons; Ce^{4+}; Ag^+; Cu^{2+}

Anionic: $[Fe^{(III)}(CN)_6]^{3-}$; $[Ir^{(IV)}Cl_6]^{2-}$; $[Ru^{(II)}(CN)_6]^{4-}$; $[Pt^{(II)}Cl_4]^{2-}$; $[Pd^{(II)}Cl_4]^{2-}$; $[Au^{(III)}Cl_4]^{-}$

[b] Aqueous soluble reductants

Neutral: $Co^{(I)}$L-H (a $Co^{(I)}$ form of vitamin B_{12}); NADH (nicotinamide adenine dinucleotide); H_2O_2

Cationic: $[Ru^{(II)}(NH_3)_6]^{2+}$

Anionic: $[Fe^{(II)}(CN)_6]^{4-}$; $[Ru^{(III)}(CN)_6]^{3-}$

[c] Organic soluble oxidants

Neutral: RBr_2 (*trans*-1,2-dibromocyclohexane); TCNQ (7,7,8,8-tetracyanoquinodimethane); TCBQ (2,3,5,6-tetrachloro-1,4-benzoquinone); TFBQ (2,3,5,6-tetrafluoro-1,4-benzoquinone); MBQ (methyl-1,2-benzoquinone); tetrachlorohydroquinone (TCHQ)

Cationic: $DcMFc^+$; $DiMFc^+$; Fc^+; $ZnPor^+$

Anionic: No example found

[d] Organic soluble reductants

Neutral: RBr_2 = *trans*-1,2-dibromocyclohexane; DcMFc (decamethylferrocene); DiMFc (dimethyferrocene); BuFc (butylferrocene); Fc (ferrocene); TTF (tetrathiafulvalene); TPTA (tri-*p*-tolylamine); ZnPor (zinc$^{(II)}$ *meso*-tetraphenylporphyrin); EDOT (3,4-ethylenedioxythiophene)

Cationic: No example found.

Anionic: $TCNQ^-$; C_{60}^- (fullerene radical anion)

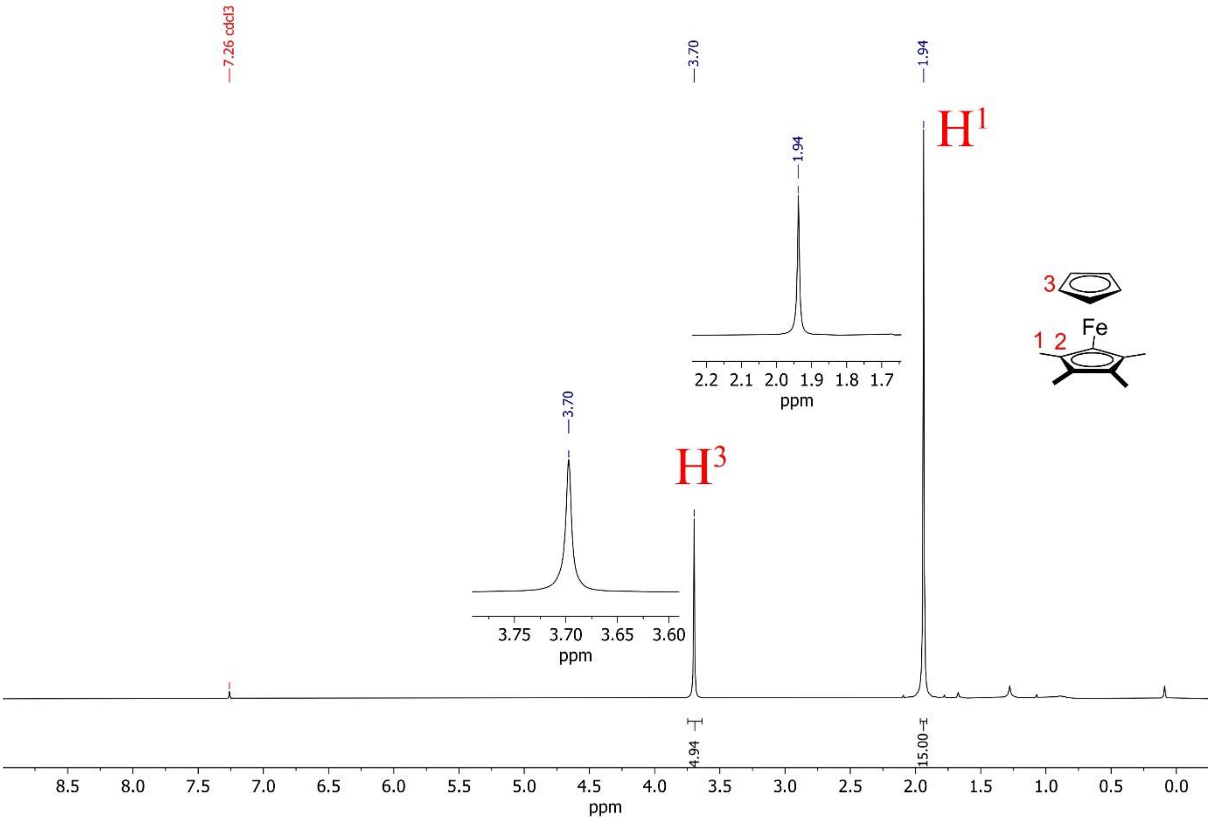

Figure S11. Plot of the ^{1}H NMR spectrum of pentamethylferrocene.

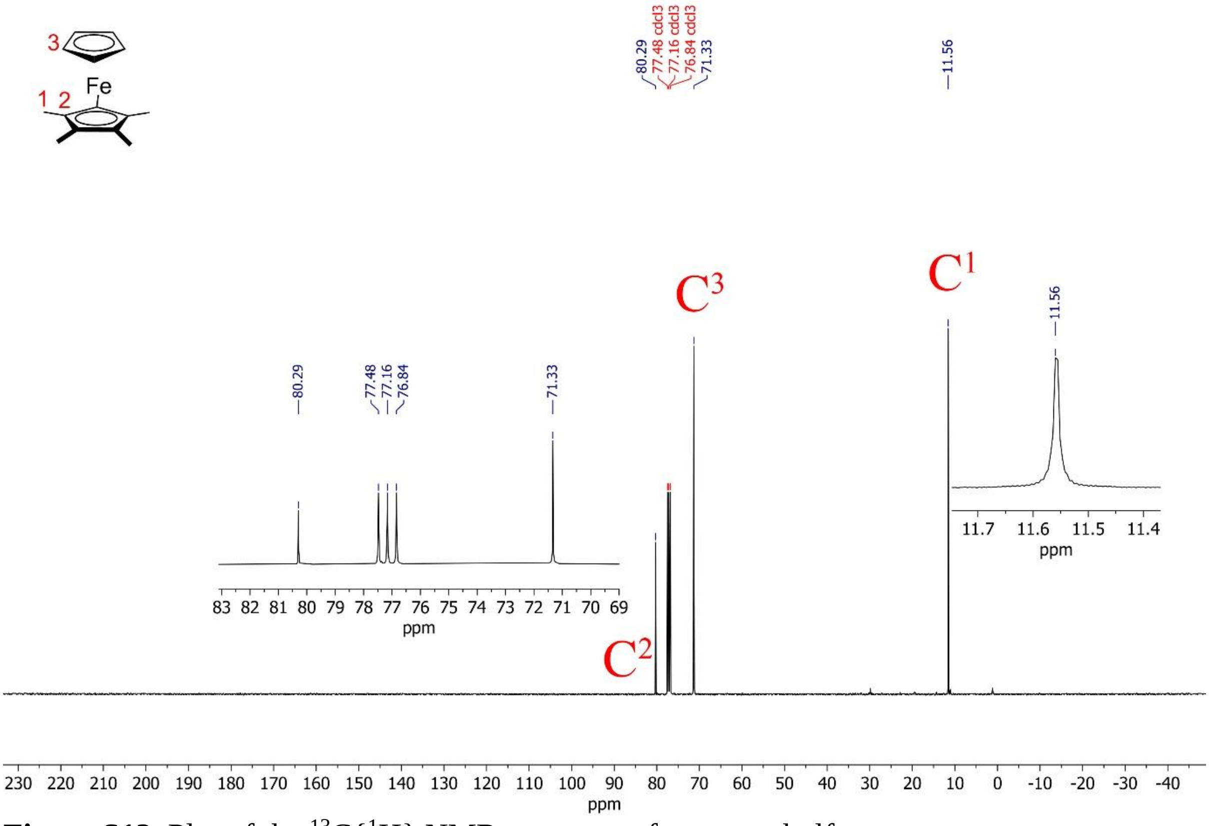

Figure S12. Plot of the $^{13}C\{^{1}H\}$ NMR spectrum of pentamethylferrocene.

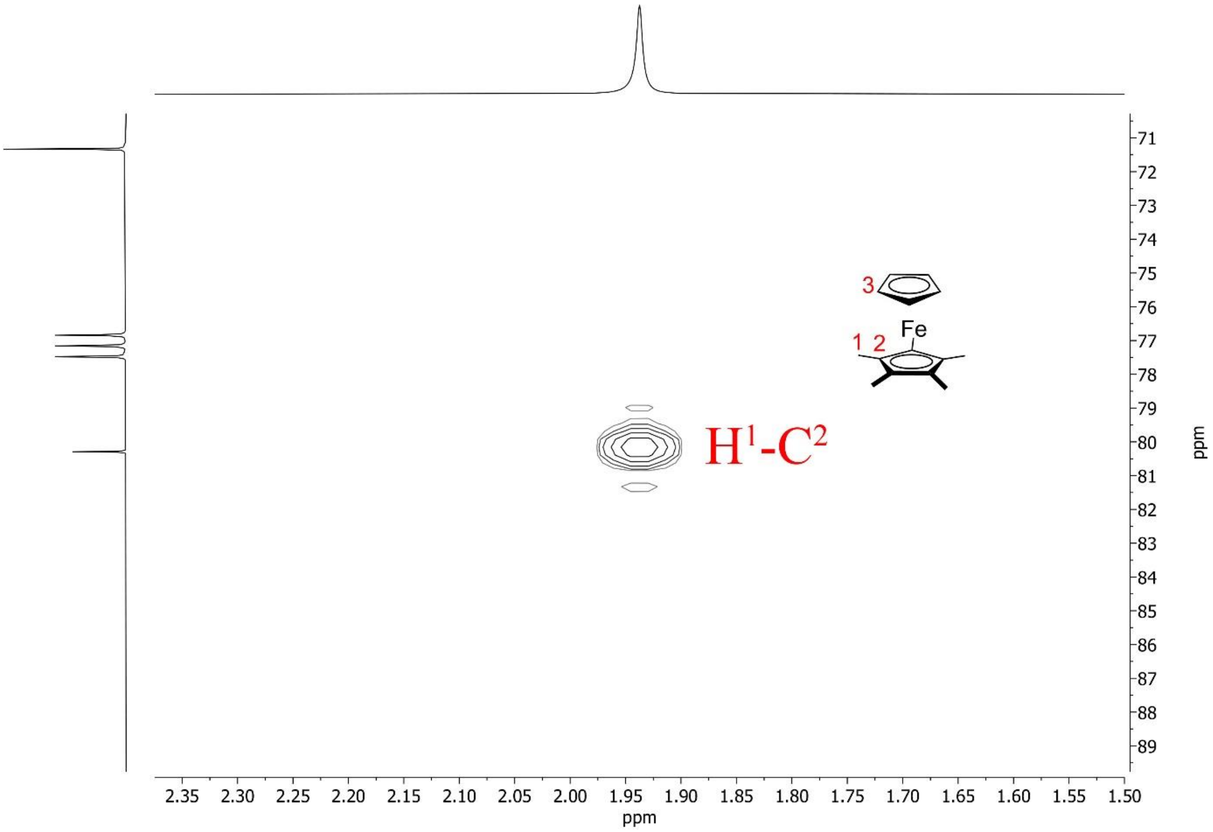

Figure S13. Plot of the ^{1}H-$^{13}C\{^{1}H\}$ HMBC spectrum of pentamethylferrocene**.**

<u>Supporting references</u>

Please see the main text article for all references.
